# Real-Time Physiological Fatigue Prediction for Human–Robot Collaborative Manufacturing Using Wearable Sensor Fusion and Hybrid Deep Learning: An In Silico Digital Twin Study

**DOI:** 10.3390/s26175556

**Published:** 2026-09-01

**Authors:** Claudio Urrea

**Affiliations:** Electrical Engineering Department, Faculty of Engineering, University of Santiago of Chile, Las Sophoras 165, Estación Central, Santiago 9170020, Chile; claudio.urrea@usach.cl; Tel.: +56-2-27183350

**Keywords:** wearable sensors, surface electromyography, fatigue prediction, machine learning, deep learning, human–robot collaboration, digital twin, in silico validation, sensor fusion, Industry 5.0

## Abstract

**Highlights:**

**What are the main findings?**

- Under leave-operators-out cross-validation on simulated data, a 73.9 k-parameter 1D-CNN–LSTM hybrid classified three fatigue states at 89.3% accuracy in 22 ms on embedded hardware, statistically indistinguishable from three larger and slower architectures.
- Ablation over fifteen feature configurations attributed 7.0 points of balanced accuracy to the sEMG block and 5.0 points to three contextual variables that require no sensor at all.

**What are the implications of the main findings?**

- What separates the models is temporal context, not architectural sophistication, so a plant can meet a 100 ms control budget without a large network.
- A four-sensor configuration retains 84.0% balanced accuracy, which makes a wearable fatigue monitor plausible at a donning cost that a work shift changeover can absorb.

**Abstract:**

Musculoskeletal fatigue precedes much of the injury burden in manufacturing, yet most monitoring schemes register an injury only once it has occurred, which becomes critical where operators share a workspace with collaborative robots. This paper presents a wearable sensing platform and a learning pipeline that track operator fatigue continuously during human–robot collaborative assembly, evaluated in silico. Eight surface electromyography (sEMG) channels, six inertial measurement units, and four force-sensitive resistors feed a 127-dimensional descriptor computed over 30 s windows. Eight classifiers were trained on 1536 simulated working hours from 24 anthropometrically diverse synthetic operators. Under leave-operators-out cross-validation, a 1D-CNN–LSTM hybrid reached 89.3% three-class accuracy and 87.1% balanced accuracy with 73.9 k parameters and 22 ms inference on a Jetson Nano; a CNN–BiLSTM–attention model gained 0.3 percentage points for 1.6 times the parameters, a difference that was not statistically significant. Ablation attributed 7.0 points of balanced accuracy to sEMG and 5.0 points to three contextual variables requiring no sensor. Alerts preceded severe fatigue by roughly 12 min, and alert-triggered task reallocation cut peak shoulder load by 43% while retaining 94% of baseline throughput. Every result characterizes a simulated environment: the study establishes internal consistency, latency feasibility, and design trade-offs, and prospective validation with human operators remains a prerequisite for deployment.

## 1. Introduction

### 1.1. Physiological Basis of Muscular Fatigue

Muscular fatigue is a progressive loss of force-generating capacity that develops whenever skeletal muscle is held under sustained or repetitive load [1]. Two families of mechanism act together, and both converge on the excitation–contraction coupling pathway. Peripherally, inorganic phosphate and hydrogen ions released by anaerobic glycolysis interfere with cross-bridge cycling at the actin–myosin filament interface; at the same time, intramuscular glycogen and phosphocreatine stores fall, leaving less substrate for adenosine triphosphate regeneration [2]. The practical consequence is a measurable drop in maximum voluntary contraction capacity. Force decrements in the order of 15–25% per hour have been reported for the kind of sustained submaximal effort typical of assembly work [3].

Central fatigue sits upstream of the neuromuscular junction. Voluntary activation, assessed by interpolated twitch, declines as excitatory drive from the motor cortex and the spinal motoneuron pool weakens [4]. The recruitment strategy changes accordingly. Higher-threshold motor units are brought in to offset losses in units already active, and the myoelectric signal registers this shift through a rise in its amplitude together with compression of the power spectrum toward lower frequencies [5]. That spectral shift remains the single most dependable surface electromyography (sEMG) marker of localized fatigue, and it is the physiological premise on which sensor-based monitoring rests [6].

Loss of force is only part of the story. Fatigued operators move differently. Joint angles vary more from cycle to cycle, trajectories lose smoothness, and posture drifts in ways that shift load from prime movers onto synergists and onto the stabilizers that assist the main muscle performing a movement [7]. Repeated over a work shift, those substitutions expose tissues to loading patterns that they are poorly configured to absorb, and this is the mechanistic bridge between acute fatigue and chronic work-related musculoskeletal disorders (WMSDs) [8]. The epidemiology is consistent with that picture: WMSDs account for a large share of the non-fatal occupational injuries and illnesses that require days away from work in the United States, and manufacturing is among the more heavily affected sectors [9].

Susceptibility to WMSDs varies widely between people, which complicates both monitoring and intervention. Body segment lengths govern mechanical advantage, body mass sets gravitational loading, and physiological cross-sectional area caps force capacity; taken together, these anthropometric factors push the inter-operator coefficient of variation in fatigue onset timing above 30% [10]. Age contributes further through sarcopenia. Fiber-type composition adds a second source of difference, this time between men and women: the higher proportion of type I fibers typically found in women favors endurance at low-to-moderate intensities, even where absolute force capacity is lower [11]. Population-average thresholds are therefore a poor basis for individual-level decisions, and some form of per-operator calibration becomes unavoidable.

### 1.2. Wearable Sensing Technologies for Physiological Monitoring

Miniaturized electronics and low-power radios have made continuous physiological monitoring practical without interfering with the task being monitored [12]. Of the available modalities, surface electromyography stays closest to the physiology of interest, since electrode pairs on the skin pick up the potentials produced when muscle fiber membranes depolarize [13]. Current wireless units sample at 1000–2000 Hz with signal-to-noise ratios above 20 dB, which is adequate for both amplitude-based activation metrics and spectral fatigue indicators once the signal has been bandpass-filtered over 20–450 Hz according to SENIAM guidance [14]. Arrays of 4–16 sites allow agonist–antagonist pairs and proximal–distal force chains to be monitored at the same time, which matters for upper-extremity assembly work [15].

Inertial measurement units (IMUs) cover what sEMG cannot, namely, the kinematics that reflects both task demand and the compensations that fatigue provokes [16]. Nine-axis units combining tri-axial accelerometers, gyroscopes, and magnetometers support drift-compensated orientation estimation through fusion filters of the Madgwick type [17]. Four to eight units distributed over the upper arm, forearm, trunk, and pelvis are enough to reconstruct joint-angle trajectories, from which range of motion, angular velocity statistics, and smoothness indices are calculated at rates compatible with on-line biomechanical analysis [18]. Bonakdar et al. compared end-to-end and feature-engineered models on IMU joint-angle data recorded during prolonged multi-activity manual handling, and they found that joint motion and inter-joint coordination alone carry usable information about performance fatigue [19].

Force-sensitive resistors (FSRs) and capacitive pressure sensors close the loop by measuring the interaction forces themselves [20]. Instrumented gloves or handle-mounted elements register how grip force is modulated, and that modulation deteriorates with fatigue: variability grows and baseline grip creeps upward as motor-unit behavior changes [21]. Once force is available alongside kinematics and sEMG, inverse dynamics yields joint torques, and torques—unlike raw sensor counts—map onto tissue-level injury criteria that ergonomists already use [22].

Bringing these modalities together in one garment is generally considered a precondition for dependable, per-operator fatigue detection. A PRISMA-guided scoping review of wearable sensing in industrial ergonomics reported that combinations of physiological and kinematic channels outperform single-channel systems, with typical gains of 5–12 percentage points in classification accuracy [23]. Babangida et al. reached a similar conclusion from the occupational-medicine side, surveying sensor systems for biomechanical risk assessment and WMSD prevention, and finding that integrated wearable architectures now approach laboratory motion capture in accuracy while remaining portable enough for a factory floor [24].

### 1.3. Machine Learning Approaches for Fatigue Classification

Turning continuous sensor streams into a usable fatigue estimate is a pattern-recognition problem in high-dimensional, noisy data [25]. Fixed-threshold rules, which declare fatigue when median frequency falls below some percentage of baseline, are simple but brittle: they ignore inter-individual variability and offer no principled way to combine channels [26]. Machine learning (ML) replaces the fixed rule with a decision boundary estimated from labeled data, and it accommodates features drawn from several physiological domains at once.

Supervised pipelines typically operate on engineered descriptors extracted from fixed-duration windows. Random Forest and gradient-boosted ensembles reach 80–87% classification accuracy when time- and frequency-domain sEMG descriptors are supplemented with kinematics [27]. Support vector machines have been applied to the same problem with comparable results: Karthick et al. classified progressive biceps brachii fatigue from high-resolution time–frequency descriptors and found that kernel machines separate fatigued from non-fatigued segments reliably once the representation is informative enough [28], while Moniri et al. combined Random Forest regression with sEMG feature forecasting to anticipate trunk muscle fatigue several seconds ahead of its occurrence [29]. Neither model family is novel in this context; both therefore appear here as baselines, with no claim of novelty attached to either. Importance analyses tend to agree on which descriptors matter: spectral compression (median frequency shift) and amplitude escalation (RMS increase) contribute most to accuracy, with postural metrics adding complementary information [30]. Evidence from actual production environments is now available as well. Mohapatra et al. instrumented 43 workers with a network of soft wearables and predicted perceived exertion—that is, each worker’s own rating of how hard the effort felt on a continuous 0–10 Borg scale—using gradient-boosted trees with an asymmetric LINEX objective and interpreting the fitted model through SHAP [31].

Deep architectures remove part of the feature-design burden by learning hierarchical representations from raw or lightly processed signals. Long Short-Term Memory (LSTM) networks are well suited to the temporal structure of fatigue, since they can distinguish a slow drift from a transient artifact by looking across successive windows [32]. Hwang et al. applied a CNN–LSTM–attention model to sEMG and IMU signals recorded from 35 participants during over-ground walking before and after a step-up-and-down fatiguing protocol, obtaining 87.94% accuracy under leave-one-subject-out cross-validation, a demanding evaluation protocol that many earlier studies avoided [26]. Hybrids that pair convolutional feature extraction with a recurrent or attentional stage have proved effective in occupational settings as well: Acharya et al. combined a Transformer–LSTM feature extractor with an XGBoost classifier for sEMG collected in simulated roofing postures, and they reported that their hybrid architecture was markedly less posture-dependent than the standard classifiers that it replaced [33]. Purely convolutional sequence models and self-attention encoders have meanwhile become standard baselines for multivariate time series in their own right [34,35,36], and both are included in the comparison reported below.

Three obstacles nevertheless persist: The first is the gap between operator-dependent and operator-independent evaluation—commonly 8–15 percentage points of classification accuracy—which reflects how much of a fitted model encodes individual physiology instead of fatigue as such [37]. The second is the move from laboratory to work floor, where electromagnetic interference, perspiration-driven impedance drift, and mechanical impacts erode 5–10% of the accuracy obtained under controlled conditions [38]. The third is latency: coupling a fatigue estimate to a collaborative robot controller imposes a budget below 100 ms, which rules out architectures whose accuracy depends on offline batch processing [39]. That figure is the one usually quoted for a controller closing a loop on the estimate that it receives; Section 3.2 states which stages of the present chain it governs and which it does not. These three obstacles cannot be addressed independently of one another, because each arises from a different stage of the processing chain. Electrode design and analog conditioning determine how much of the physiological signal survives the factory environment, window length and feature definition determine how much individual physiology is encoded, and network depth determines how much latency the inference stage consumes. A decision taken at any one stage constrains the others, so the sensor hardware, the conditioning chain, the feature set, and the model architecture have to be designed jointly.

### 1.4. Research Gaps and Contributions

Wearable sensing and machine learning are each mature enough for fatigue monitoring. What is still scarce is a system in which the two are combined, run in real time, and tested against the specific demands of human–robot collaborative (HRC) manufacturing. Three gaps stand out:

Most multi-modal fusion studies still rely on laboratory protocols with healthy young volunteers performing isolated contractions [40], so little is known about behavior under multi-joint movement, fluctuating task demand, and shift-length exposure. Systematic comparisons are also rare. A single preferred architecture is usually pitted against one weak baseline on one dataset, which says little about where each family of models is actually strong [19]. Finally, end-to-end operation has not been reported at scale: acquisition, fusion, feature extraction, inference, and intervention triggering, all inside a latency budget that a cobot controller can tolerate [31].

Four contributions address these gaps; all four are established in silico (see Section 6): the sensing hardware is specified at the component level, and its output is emulated by a biomechanically calibrated digital twin, so that validation covers the design of the pipeline but stops short of its behavior on human tissue.

*C1: Multi-Modal Wearable Platform:* A sensing architecture of eight sEMG channels, six IMUs, and four force-sensitive resistors (FSRs), laid out for unobtrusive wear during collaborative assembly, together with the conditioning chain that each modality requires. Both were exercised against synthetic signals produced by the digital twin.

*C2: Feature Set:* A 127-dimensional descriptor spanning time-domain, spectral, kinematic, force, and model-derived biomechanical quantities, computed over 30 s windows so that slow physiological drift and within-cycle detail are both retained. A modality ablation quantifies what each block contributes and what a reduced instrumentation set would cost in performance.

*C3: Model Comparison and Hybrid Architecture:* Eight classifiers, from Random Forest and a support vector machine through XGBoost to a temporal convolutional network, a Transformer encoder, an LSTM, a 1D-CNN–LSTM hybrid architecture, and a CNN–BiLSTM–attention model, trained and tested on identical partitions under leave-operators-out cross-validation, and compared in terms of accuracy, parameter count, arithmetic cost, and measured edge latency.

*C4: Intervention Effect:* Digital twin trials over 24 synthetic operators and 1536 simulated working hours, quantifying what alert-triggered task reallocation buys: 43% lower peak shoulder moment against a 6% throughput cost.

Every result reported here comes from simulation, and the consequences of that are stated at the outset rather than left to the limitations section. No human participant took part at any stage, and no physiological signal was recorded from a person. The sEMG, inertial, and force streams were synthesized from the biomechanical state of a musculoskeletal model through the transduction and noise models of Section 6.4, and the fatigue labels are the exact internal state of that model, not a proxy measurement or a self-report. What this design can settle is whether the pipeline runs end-to-end within a stated latency budget, how the eight architectures rank on identical data, and what trade-offs hold between accuracy, sensor count, and threshold placement. What it cannot settle is whether any of the resulting figures transfers to a person. The accuracy, the 12 min lead time, and the 43% load reduction are properties of this simulation and inherit every assumption of the model that generated them, and a prospective study with human operators remains a prerequisite for deployment. Section 2.4 places the limitation in the context of the human digital twin literature, and Section 8.4 works through its consequences.

Section 2 reviews prior work. Section 3 specifies the sensing platform, placement, and conditioning chain. Section 4 covers feature engineering, the eight architectures, and the training protocol. Section 5 describes the musculoskeletal model and the personalized fatigue dynamics that supply the ground truth. Section 6 sets out the digital twin and the experimental design. Section 7 reports results, and Section 8 and Section 9 discuss them and present the conclusions. Section 3, Section 4, Section 5 and Section 6 are written for readers who intend to reproduce or adapt the system and are correspondingly detailed; a reader interested only in what the system achieves and what it does not can move from here to Section 7 and Section 9 without loss of continuity.

## 2. Related Work

### 2.1. Physiological Fatigue Monitoring in Occupational Settings

Over two decades, occupational fatigue assessment has moved from self-report toward instrumented measurement. The early instruments were questionnaires, chiefly the Borg Rating of Perceived Exertion (RPE) scale [41] and the NASA Task Load Index (NASA-TLX) [42]. Both capture global perception well, but neither offers the temporal resolution that an intervention system needs. Observational tools such as the Rapid Entire Body Assessment (REBA) and the Rapid Upper Limb Assessment (RULA) added systematic postural scoring. Their reliability, however, is limited by the human observer. The relevant property is inter-rater reliability, conventionally expressed as an intraclass correlation coefficient (ICC), which reports how much of the total variance in the scores is attributable to the tasks being rated rather than to the raters themselves. When several trained raters score the same recorded task, the ICC between their REBA scores falls in the range 0.68–0.82, read by convention as moderate-to-good agreement [43]. Agreement at that level is sufficient for a periodic ergonomic survey, where the quantity of interest is a group average, but not for an automated decision taken every few seconds inside a safety-critical control loop, where two equally qualified assessors would sometimes disagree on the score that triggers the intervention.

Instrumented assessment began in the laboratory, with sEMG studies relating sustained contraction to compression of the power spectrum, a relationship documented in the surface electromyography literature well before wearable hardware existed [6]. Transfer to occupational settings has accelerated with wearable hardware. Mohapatra et al. monitored 43 workers performing composite layup and wire harnessing with a soft, skin-interfaced sensor network, treated fatigue as a continuous rather than a dichotomous variable, and showed that an asymmetric loss function reduces the under-predictions of exertion that matter most when the output feeds a safety decision [31]. Bonakdar et al. worked from kinematics alone, extracting joint angles and inter-joint coordination from IMUs during prolonged manual handling and comparing end-to-end against feature-engineered models [19]. Related lines of work point in the same direction, among them fatigue-aware task planning driven by a human digital twin [44] and fatigue classification under varying thermal load [45]; a recent survey traces the convergence of wearable sensing with automated decision-making in some detail [46].

Cardiac measures offer a second, autonomic view of the same process: heart rate variability (HRV) indexes the balance between sympathetic and parasympathetic outflow of the autonomic nervous system, and that balance shifts when physical and cognitive fatigue accumulate [47]. Khan et al. combined electromyographic and cardiac features for construction workers under thermal stress and classified three levels of physical fatigue with 80–90% accuracy, which suggests that multi-modal descriptors hold up reasonably well when ambient conditions change [45]. Cardiac sensing was nonetheless left out of the platform described below, for two reasons: HRV responds to thermal load, hydration, and emotional state as strongly as to mechanical exposure; and a chest-worn electrocardiogram unit adds a donning step whose cost is examined in Section 8.7. Workplace trials with low-cost sEMG hardware have separately confirmed that sensor-derived fatigue metrics track subjective exertion during manual handling without any cardiac channel [48].

### 2.2. Wearable Sensors in Manufacturing Environments

A factory imposes problems that a laboratory does not. Welding gear, motors, and power electronics radiate interference that corrupts sEMG unless hardware shielding and adaptive filtering are in place [49]. Impacts, vibration, and perspiration act on the sensor–skin interface itself, generating motion artifacts that contaminate sEMG and IMUs alike. The SENIAM recommendations on electrode placement and processing mitigate much of this, provided they are followed to the letter [14].

Miniaturization has nonetheless made industrial deployment realistic. The scoping review cited above notes that systems built from a small number of modalities strike a better balance between coverage and wearer compliance, since every additional sensor is one more thing that an operator can find intrusive [23]. Babangida et al., reviewing sensor systems for biomechanical risk assessment, reported that integrated wearable architectures now come close in accuracy to laboratory motion capture for upper-extremity movements of the kind that assembly work involves, while remaining portable [24].

Collaborative workspaces add further constraints. Proximity to robot actuators raises the interference level, so differential sEMG with common-mode rejection above 100 dB stops being a refinement and becomes a requirement [50], and no part of the instrumentation may impede natural movement or intrude on the separation distances that ISO/TS 15066 specifies for collaborative operation [51]. Empirically, Wu et al. instrumented sixteen participants with Xsens motion capture and Trigno sEMG during manual and cobot-assisted agricultural transplanting. Cobot assistance lowered segment velocity and acceleration at vertebrae L5/S1, L1/T12, and T1/C7, and at the same time redistributed muscle activation away from the biceps brachii and toward the flexor carpi radialis, brachioradialis, and upper trapezius. Robotic assistance therefore changes the anatomical distribution of the load—not only its magnitude—and a monitoring system that observes only the muscles that are unloaded by the cobot will report an improvement that the operator does not experience [52].

### 2.3. Machine Learning for Fatigue Classification

Work in this area runs along a continuum from hand-crafted features to end-to-end learning. Reviews of sEMG-based fatigue assessment place conventional classifiers operating on time-domain descriptors (RMS amplitude, mean absolute value, zero crossings) together with spectral ones (median frequency, mean power frequency) at roughly 75–85% correct classification for binary decisions [53]. Adding temporal context lifts that figure, for the straightforward reason that fatigue is a trajectory and a single window is a snapshot of it.

LSTMs have served this purpose well. Wang et al. paired an LSTM with improved wavelet packet threshold denoising for sEMG fatigue classification and gained 6–10 percentage points of classification accuracy over static classification of the same features [54]. Hybrids have since pushed further; Zhao et al., classifying motion states in an upper-limb human–exoskeleton system, combined convolutional local feature extraction with bidirectional recurrence and attention weighting [55]. Outside the fatigue literature, dilated causal convolutions [34] and self-attention encoders [35] have become the default reference points for multivariate sequence modeling [36], and any claim that a recurrent hybrid is preferable now has to be argued against them, not against classical models alone.

Explainability has become a practical requirement, not an academic nicety, because an ergonomist who cannot see why an alert fired will not act on it. SHAP (SHapley Additive exPlanations) supplies model-agnostic attributions that let a domain expert check whether a learned boundary corresponds to anything physiological [56,57]. Applied to gradient-boosted models in occupational settings, such attributions have placed cardiac descriptors and limb kinematics near the top of the ranking, in patterns consistent with what ergonomics already predicted [31].

### 2.4. Digital Twins and Human Digital Twins in Manufacturing

A digital twin is a computational model of a physical asset that is kept in correspondence with it, so that the model can be simulated forward in time and its predictions used to decide what the asset should do next. Applied to a production cell, the asset is the cell itself, and the twin reproduces robot kinematics, part flows, and cycle times. Applied to an operator, the asset is a person, and the model has to represent anthropometry, joint kinematics, muscle force capacity, and the way that capacity declines under load. This second variant, usually called a human digital twin, is the one that this paper relies on, and three of its properties matter for what follows:

The first property is that a human digital twin is mechanistic in construction: its behavior follows from equations of motion and from a physiological state that the model itself advances, with no fitted statistical component interposed. Its internal state, and in particular the accumulated activation of each muscle, is available exactly at every instant of the simulation, whereas in a human experiment that state can only be inferred from an sEMG proxy or reported on a subjective scale. This is what makes a twin usable as a source of labels. The second property is that a twin can be driven repeatedly into loading regimes that an ethics committee would not approve for a human participant, so the regions of the state space where a fatigue predictor most needs testing are exactly the regions that a twin can reach easily. The third property is the corresponding weakness: the labels that a twin produces inherit every assumption of the model that produced them, so a fatigue predictor trained against them is validated against a hypothesis about fatigue, never against fatigue itself. This applies to the present study in the strongest form that the literature offers, because the twin here supplies not only the labels but the sensor signals as well, so nothing in the measurement chain is anchored to a physical recording. Agreement between the classifier and the twin-derived label is therefore evidence about the classifier and not about human physiology, and whether any figure in Section 7 would survive contact with a factory is untested here. Section 7.7 and Section 8.4 return to this point in detail, because it delimits every claim made in this paper.

The infrastructure for the first variant is established. Baratta et al. set out the simulation architecture required to represent human–robot collaboration in manufacturing systems [58], and Vongchaisaree and Abdoli formalized the human digital twin as an instrument of physical hazard management, mapping the components that a hazard-oriented twin must include [59]. Two applications are closer to the present work: You et al. built a human digital twin that estimates physical fatigue in real time and feeds a task planner that reallocates assembly subtasks accordingly, validating the arrangement in a multi-subject assembly experiment [44]; the fatigue state there was estimated from a biomechanical model driven by tracked motion. Chand et al. took a vision-based route to the same objective, monitoring physical and cognitive fatigue non-invasively within a human-centric HRC framework and testing it on a bookshelf assembly performed by a human–robot team [60]. In both cases, the twin supplies the state estimate that a controller acts on, which is the role that it plays here as well, with one difference: in this study the twin also generates the sensor signals from which that estimate is learned, so the entire measurement chain can be exercised before any hardware is procured.

## 3. Multi-Modal Wearable Sensor Platform

### 3.1. Sensor Hardware Specifications

Three modalities were selected so that neuromuscular activation, segment kinematics, and contact force were each observed directly, none of them inferred from the others. Table 1 lists the hardware. The specifications given here define the target instrument: they fix the sampling rates, resolutions, noise levels, and packet-loss behavior that the synthetic signal generator of Section 6.4 reproduces, so that the pipeline is developed against the hardware that it is intended to run on.

*Surface Electromyography:* Eight wireless Trigno Avanti sensors (Delsys Inc., Natick, MA, USA) cover four muscles bilaterally: the biceps brachii (elbow flexion), anterior deltoid (shoulder flexion and abduction), flexor carpi radialis (wrist flexion and grip), and upper trapezius (shoulder elevation and stabilization). Placement follows SENIAM [14]. The measurement configuration uses bipolar 10 mm Ag/AgCl electrodes at 20 mm spacing and rejects common-mode signal by more than 100 dB; below that figure, the arrangement would be unusable near powered actuators. Sampling is at 1000 Hz with 16-bit resolution, giving 1 μV sensitivity over the 20–450 Hz acquisition band.

*Inertial Measurement Units:* Current Trigno Avanti sensors embed a nine-axis inertial unit alongside the electromyographic front end, so in principle the kinematic channels could have been taken from the same eight devices. They were not, for three reasons: The embedded unit samples accelerometry at 148 Hz but streams orientation at a reduced rate. It is also bound to the electrode sites, which were chosen to sit over muscle bellies, a criterion unrelated to segment tracking. Finally, the sacrum and sternum, which anchor the trunk and pelvis segments of the musculoskeletal model, carry no electrode at all. A dedicated inertial system therefore decouples the two placement problems. Six MTw Awinda units (Xsens Technologies B.V., Enschede, The Netherlands) sample a tri-axial accelerometer (±16 g), gyroscope (±2000° s^−1^), and magnetometer at 100 Hz [61], and they are mounted on the bilateral upper arm, bilateral forearm, sternum, and sacrum. Orientation is reported as quaternions, which keeps joint-angle reconstruction free of gimbal singularities [62]; the on-board Madgwick filter (β=0.1) fuses the three signals and holds static accuracy below 1° RMS [17]. A single-vendor configuration built on Trigno Avanti alone is a legitimate alternative for a cost-constrained deployment, and Section 8.7 quantifies what the reduced kinematic coverage would cost in balanced accuracy.

*Force-Sensitive Resistors:* Four FlexiForce A201 elements (Tekscan, Inc., Norwood, MA, USA) measure interaction force. None of them is worn: two are bonded to the handle of the dominant-hand power tool, one to the jaw of the assembly gripper, and one to the workstation support surface, so the operator contacts them only through the objects being handled. The 0–110 N range at 0.5 N resolution and 500 Hz is sufficient to resolve grip modulation during fastener installation, part retrieval, and inspection [63].

### 3.2. Sensor Placement and Data Acquisition Architecture

**Figure 1:**
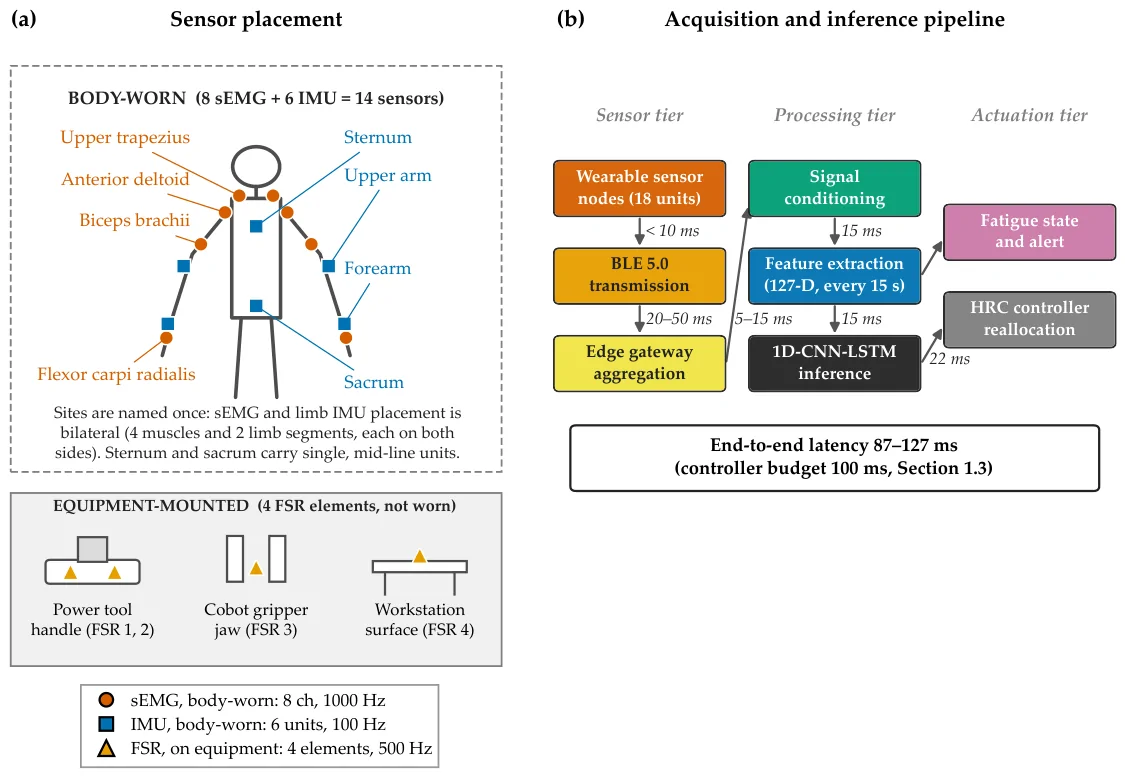
Wearable sensor platform configuration and real-time data acquisition pipeline. (**a**) Sensor placement. The upper region contains the 14 body-worn devices: 8 sEMG channels (circles) on four upper-extremity and trunk muscles taken bilaterally, and 6 IMU units (squares) on the bilateral upper arm and forearm together with single mid-line units at the sternum and sacrum. Each site is named once, since sEMG and limb IMU placement is symmetric. The shaded lower region contains the 4 FSR elements (triangles), which are not worn: two are bonded to the power tool handle, one to the cobot gripper jaw, and one to the workstation surface, and the operator contacts them only through the objects being handled. (**b**) Three-tier data acquisition architecture from wireless sensor nodes through BLE 5.0 transmission and edge gateway aggregation to server-side signal processing, feature extraction, and ML inference, yielding an end-to-end latency of 87–127 ms, compatible with the timescale on which fatigue state evolves.

Figure 1 illustrates the sensor placement configuration and the data acquisition pipeline. The acquisition architecture employs a three-tier topology designed to minimize latency while maintaining data integrity.

Each of the 18 sensors timestamps its samples in hardware against a shared synchronization beacon and transmits over Bluetooth Low Energy (BLE 5.0) [64]. The gateway collects the 66 raw channels that these 18 units produce, aligns them, and forwards the aligned packets over IEEE 802.11ac [65]. Alignment proceeds in two stages, and both run on-line: the hardware timestamps place all streams on a common clock, and a residual correction is then applied by cross-correlating shared mechanical events, since a movement onset produces a simultaneous sEMG burst and an IMU acceleration transient. The residual correction is needed because the three radios do not incur identical transmission delays, and it operates on a rolling 10 s buffer, which adds latency but requires no post-processed labels and no operator intervention; Section 3.3 gives the resulting skew. Conditioning, feature extraction, and inference run on the server, which returns the fatigue estimate to the HRC controller.

Three further variables reach the same feature vector without passing through this chain. Elapsed time since work shift start, elapsed time since the last rest break, and cumulative cycle count are read from the manufacturing execution system (MES) that already schedules the cell, over the same IEEE 802.11ac link, at the 15 s cadence of the feature extractor. Being counters, they carry no waveform to align and need no synchronization finer than one analysis window; the MES timestamp is simply matched to the window boundary. They are listed as the contextual block in Table 2 and analyzed in Section 7.4 and Section 7.5.

The latency budget breaks down as follows: digitization and BLE transmission below 10 ms, gateway aggregation and synchronization 20–50 ms, WiFi relay 5–15 ms, conditioning 15 ms, feature extraction 15 ms, and inference 22 ms, for 87–127 ms end-to-end.

The upper end of that interval exceeds the 100 ms figure quoted in Section 1.3. The two numbers do not measure the same thing. The 100 ms figure is what a collaborative robot controller can tolerate between receiving an estimate and acting on it, and the stage of the present chain that competes for it is inference, which takes 22 ms for the hybrid and stays below 40 ms for all eight architectures in Section 7.2. The 87–127 ms interval covers the whole path from transduction to an estimate arriving at the controller, most of which is transport and buffering: gateway aggregation and synchronization account for 20–50 ms and the WiFi relay for a further 5–15 ms, so the worst case arises only when both are simultaneously at maximum, and none of the excess is attributable to the model.

The more substantive point is that the fatigue channel is not a reactive control loop and was never designed as one. A new estimate is issued once every 15 s, the state that it tracks evolves over minutes, and the alert that it raises precedes severe fatigue by roughly 12 min; the 27 ms by which the worst case overruns 100 ms is more than four orders of magnitude smaller than that margin, and no decision in this system is sensitive to it. Where an integrator nevertheless imposes a hard bound below 100 ms, two measures are available without retraining: hosting inference on the gateway removes the WiFi relay, and the INT8 quantization reported in Section 7.2 reduces inference from 22 to 14 ms, which together bring the interval to 74–104 ms. What cannot be served by this path on any budget is collision avoidance, which needs sub-10 ms response and must remain on a separate, dedicated channel; the fatigue estimate is advisory input to task allocation, not a safety function.

### 3.3. Signal Processing Pipeline

**Figure 2:**
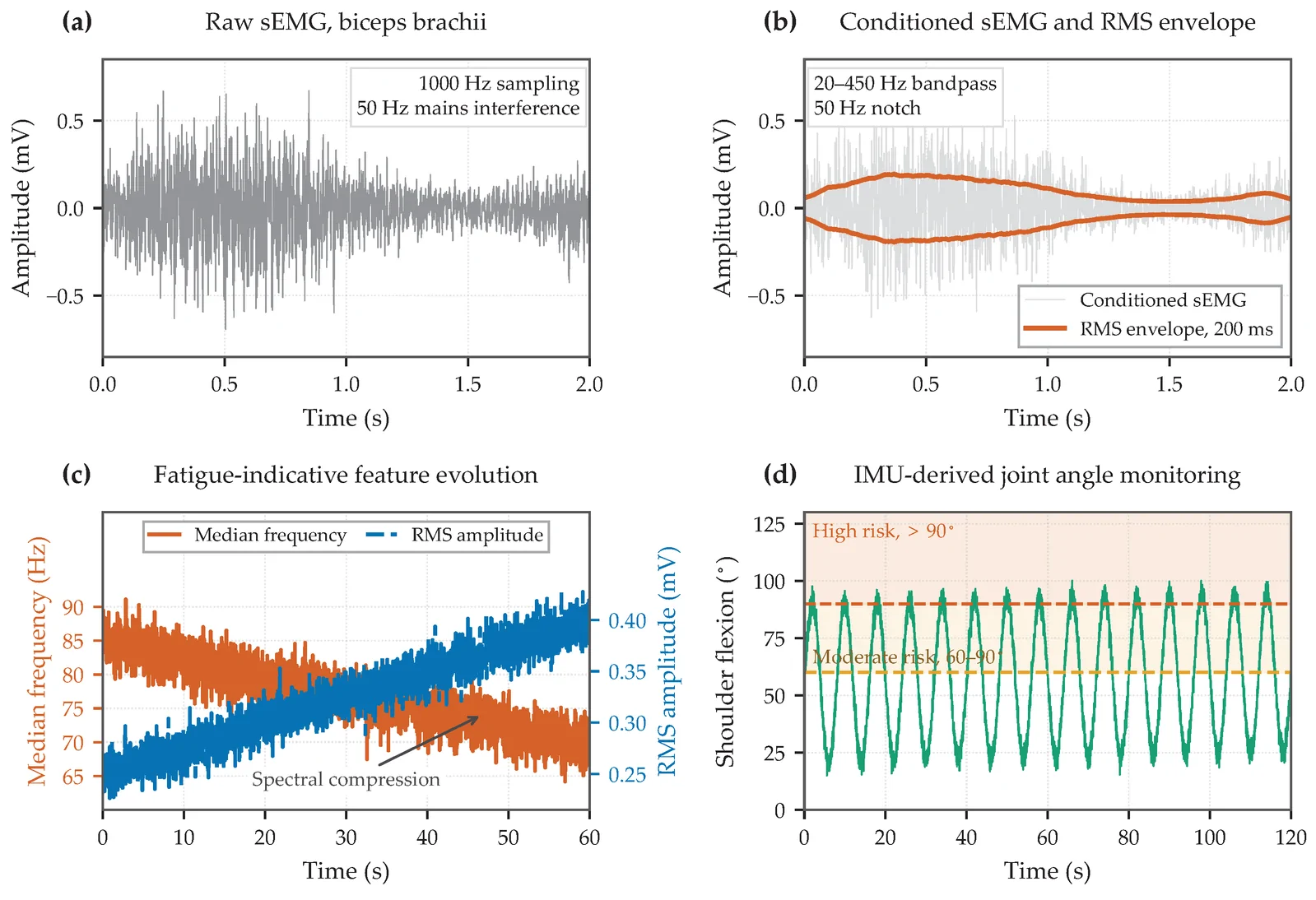
Signal processing pipeline illustrated through representative waveforms from the multi-modal sensor platform. (**a**) Raw sEMG signal from the biceps brachii channel contaminated with 50 Hz power line interference and broadband noise at 1000 Hz sampling. (**b**) Bandpass-filtered (20–450 Hz) sEMG output with overlaid 200 ms RMS envelope capturing activation intensity dynamics. (**c**) Fatigue-indicative feature evolution over a 60-s sustained contraction showing the characteristic inverse relationship between declining median frequency (left axis) and increasing RMS amplitude (right axis); the arrow marks the region of steepest spectral compression, that is, the interval over which the median frequency falls most rapidly. (**d**) IMU-derived shoulder flexion angle during repetitive assembly cycles, with ergonomic risk thresholds annotated at 60° (moderate) and 90° (high risk).

Each modality is conditioned separately, the aim being to remove artifacts without eroding the physiological content on which the features depend. Figure 2 traces the chain from raw acquisition to feature-ready signals.

*sEMG:* A fourth-order Butterworth bandpass (20–450 Hz) removes sub-20 Hz motion artifacts and supra-450 Hz noise while leaving the physiologically relevant band intact [66], and a second-order IIR notch at 50 Hz with 2 Hz bandwidth suppresses mains interference. Full-wave rectification followed by a 200 ms RMS envelope gives the activation-intensity estimate used for amplitude features. Spectral analysis runs in parallel on the bandpassed signal, where a short-time Fourier transform with 500 ms Hamming windows at 50% overlap yields median and mean power frequency trajectories. Since assembly involves cyclic dynamic contractions rather than sustained isometric holds, this time–frequency treatment is necessary; a single spectrum computed over the whole window would violate the stationarity that classical fatigue indices assume [67].

*IMU:* Quaternion orientations from the on-board filter drive kinematic reconstruction. Joint angles follow from relative quaternions between adjacent segments [68]: shoulder flexion/extension and abduction/adduction from trunk to upper arm, elbow flexion/extension from upper arm to forearm, and trunk orientation from pelvis to sternum. No sensor is placed on the hand, so wrist angles are not measured directly. They are instead resolved by the inverse-kinematics solver of Section 5 under a minimum-deviation constraint. This distinction is drawn explicitly because it bounds what the biomechanical features can represent: shoulder and elbow quantities rest on measured orientations, whereas wrist quantities rest on an assumption about how the solver distributes residual motion, and they should be given correspondingly less weight.

Accelerations are measured in the local reference frame of each sensor, which moves with the segment to which it is attached. Once the segment orientation is known, the translational acceleration is rotated into a fixed global frame, in which the direction of the gravitational vector is known and can be subtracted, leaving the acceleration due to voluntary movement alone. That signal is then low-pass filtered with a fifth-order Butterworth at 5 Hz, which separates voluntary movement from vibration and impact. Jerk is obtained by Savitzky–Golay differentiation (n=3, frame length 11 samples), which differentiates and smooths in one operation and, thus, avoids amplifying the noise [69].

*FSR:* Piezoresistive elements are markedly nonlinear, so each channel is linearized against a calibration curve acquired at system initialization [63]. A five-sample median filter removes impulsive noise without blurring force onsets and offsets. Any interval in which measured force reaches the 110 N ceiling is flagged as saturated and annotated for the feature extraction stage.

*Synchronization:* The residual alignment stage introduced in Section 3.2 brings inter-modality skew below 5 ms [70]. The aligned streams are then cut into 30 s windows with 50% overlap, so a new feature vector is produced every 15 s.

### 3.4. Feature Engineering

Every 30 s window yields a 127-dimensional vector. Table 2 gives the breakdown.

*sEMG (64 dimensions, 8 per channel):* Five time-domain descriptors characterize activation intensity and signal complexity: root mean square (RMS) amplitude, mean absolute value (MAV), waveform length (WL), zero crossings (ZC) and slope sign changes (SSC) [25,71]. Three frequency-domain descriptors complete the set, namely, median frequency (MDF), mean power frequency (MNF), and a fatigue index

(1) $${\mathrm{FI}}_{i}\left(t\right)=\frac{{\mathrm{MDF}}_{i}\left(t_{0}\right)-{\mathrm{MDF}}_{i}\left(t\right)}{{\mathrm{MDF}}_{i}\left(t_{0}\right)},$$

where ${\mathrm{MDF}}_{i}\left(t_{0}\right)$ is the session baseline for channel *i*. Normalizing against each channel’s own baseline in this way removes the between-channel amplitude differences that arise from electrode placement and subcutaneous tissue thickness, leaving a quantity that is comparable across muscles and across operators. Spectral compression is the primary electrophysiological fatigue indicator, so all three of these descriptors carry the same underlying signal in different forms [6].

*IMU (36 dimensions, 6 per unit):* Mean joint angle, mean and standard deviation of angular velocity, range of motion within the window, dimensionless normalized jerk, and acceleration variability. Normalization matters here, because raw jerk scales with movement duration and amplitude, which would confound a comparison between operators working at different rates [72]. Collectively, these descriptors register what fatigue does to movement: more variability, less smoothness, and postural drift that shifts load away from the muscles doing the work.

*Biomechanical (12 dimensions):* Inverse dynamics (Section 5) supplies peak and cumulative shoulder torque, peak and cumulative elbow torque, peak grip force, and mechanical work, each computed bilaterally. These twelve quantities are the only ones in the vector expressed in units that an ergonomist can compare against an exposure limit, so they are retained even though they are derived from the remaining 115.

*FSR (12 dimensions, 3 per element):* Peak force, force variability expressed as a coefficient of variation, and force rate of change. Grip control degrades in a characteristic way as the finger flexors fatigue: variability rises and baseline grip creeps upward, the latter apparently to guard against dropping the object [21].

*Contextual (three dimensions):* Elapsed time since work shift start, elapsed time since the last rest break, and cumulative cycle count, obtained from the MES as described in Section 3.2. None of these requires a sensor, yet they encode the accumulation that instantaneous physiological measurements cannot.

Standardization to zero mean and unit variance is performed per operator, using statistics computed from that operator’s baseline period only. This absorbs the large between-operator differences in absolute signal magnitude while leaving the within-operator temporal dynamics untouched; those dynamics are where the fatigue information resides.

## 4. Machine Learning Fatigue Prediction Pipeline

### 4.1. Dataset Characteristics and Fatigue Labels

The dataset comes from the digital twin of Section 6: 24 synthetic operator models, 8 sessions each, 192 sessions in total, and 1536 operational hours. The cohort specification, the simulation configuration, and the seeds that regenerate it are deposited [73]. A session covers an 8 h work shift with the usual rest breaks.

Ground truth is the Fatigue Demand Index (FDI), a composite score on a 0–100 scale computed from the biomechanical model of Section 5 as the cumulative activation of the eight instrumented muscles, each normalized by its own maximum isometric capacity and integrated over elapsed time under load. The FDI is thus an internal state variable of the simulation, not a quantity estimated from the synthetic sensor signals, and it is this that makes the labels exact within the simulation. It is not a measurement: Section 7.7 examines how far it behaves like the physiological fatigue indicators for which it is meant to stand, and Section 8.4 sets out what follows for the interpretation of every result in this paper.

Three classes are defined by two thresholds on FDI:

- **Fresh** (FDI ≤40): Low cumulative loading, full force capacity available.
- **Moderate** (40< FDI ≤70): Elevated loading with measurable performance decrements.
- **Severe** (FDI >70): High loading approaching injury risk thresholds.

The two cut-points were fixed before any model was trained, on three grounds: First, the fatigue model of Equation (5) maps FDI onto retained force capacity, and FDI = 40 corresponds to a mean loss of 11.2% in initial maximum voluntary contraction (MVC) capacity across the cohort, while FDI = 70 corresponds to a loss of 26.8%. The upper figure sits inside the 15–25% hourly decrement range reported for sustained submaximal upper-limb work [3], so the severe class begins where measured force capacity has fallen by about a quarter. Second, the endurance-limit literature places the boundary of indefinitely sustainable static effort near 15–20% MVC and treats accumulated exposure above that level as requiring a rest allowance [74,75]; expressed as cumulative normalized activation over an 8 h work shift, the corresponding FDI values are 38 and 72. Third, a receiver operating characteristic analysis performed directly on the simulated force-capacity trajectories, taking a 10% and a 25% capacity loss as reference events, gave Youden-optimal FDI cut-points of 38.6 and 71.4. The rounded values 40 and 70 were adopted for legibility. Because all three arguments rest on the model itself and none of them is empirical, Section 7.6 reports how classification performance, severe-class recall, and alert lead time respond when this pair of FDI cut-points is moved.

Windowing accounts for the sample count as follows: Each 8 h session contains 60 min of scheduled breaks, leaving 7 h of task time, or 1680 windows at the 15 s stride. Windows straddling a break boundary are discarded, as are those carrying a saturation or dropout flag from the conditioning stage; together, these remove about 8.6% of the total and leave 1536 windows per session—hence, 12,288 samples per operator and 294,912 samples across the cohort. The resulting class distribution is approximately 45% fresh, 38% moderate, and 17% severe. This is a property of the simulated work shift structure and of the fatigue-effect formulation driving it [76], not an empirical prevalence estimate, and it should not be read as one.

### 4.2. Model Architecture Comparison

Eight architectures were trained on identical partitions, so this paper compares the models on the same data. Classical models use scikit-learn (https://scikit-learn.org, accessed on 30 August 2026) [77], while the neural models use PyTorch (https://pytorch.org, accessed on 30 August 2026) [78]. The first three classify each window in isolation; the remaining five see a sequence of six consecutive feature vectors, which at a 15 s stride and 30 s windows spans 105 s of elapsed time.

*Random Forest (RF):* Two hundred trees, maximum depth 15, $\lfloor \sqrt{127}\rfloor =11$ features considered per split, majority vote across trees.

*Support Vector Machine (SVM):* Radial basis function kernel, C=10, γ=0.01, one-versus-rest for the three classes, with Platt scaling applied to obtain calibrated probabilities [79].

*Gradient-Boosted Trees (XGBoost):* Implemented with the XGBoost library (https://xgboost.ai, accessed on 30 August 2026). Three hundred trees, learning rate 0.05, maximum depth 8, L2 coefficient 1.0, early stopping on validation loss [80].

*Temporal Convolutional Network (TCN):* Four residual blocks of dilated causal convolutions, 64 channels, kernel size 3, and dilation factors 1, 2, 4, and 8, giving a receptive field that covers the full six-window sequence, with weight normalization and dropout 0.2 inside each block [34]. The TCN is included because it reaches the same temporal span as a recurrent model without maintaining a recurrent state, and a deployment engineer would consider that alternative first.

*LSTM:* Two layers of 128 and 64 hidden units, dropout of 0.3 between layers, a dense softmax output, Adam at learning rate $10^{-3}$, and categorical cross-entropy [81].

*Transformer Encoder:* Two encoder layers with model dimension 128, four attention heads, feed-forward dimension 256, learned positional encoding, and dropout 0.1, followed by mean pooling over the sequence and a softmax head [35,36]. Six positions is a short sequence for self-attention, and this model is included to establish whether that limitation is decisive at the sequence length that the application imposes.

**Figure 3:**
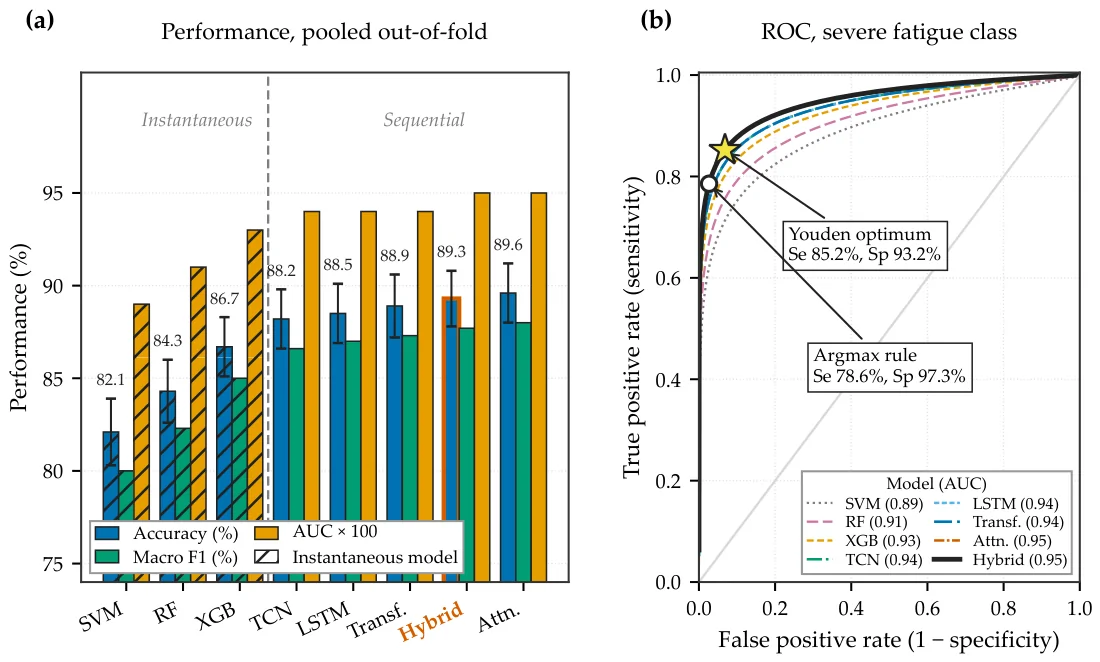
Classification performance comparison across eight model architectures. (**a**) Bar chart of accuracy, F1-score, and AUC for each architecture; the three instantaneous models are shaded separately from the five sequential ones, and the 1D-CNN–LSTM hybrid is outlined. (**b**) Receiver operating characteristic curves computed one-versus-rest for the severe fatigue class. Curves are binormal fits constrained to reproduce each model’s AUC and, for the hybrid, to pass through its argmax operating point (sensitivity 78.6%, specificity 97.3%) implied by the confusion matrix reported in Section 7.3. The star marks the Youden-optimal point of the hybrid curve (sensitivity 85.2%, specificity 93.2%). It differs from the severe-class recall reported in Section 7.3 because that value follows from the argmax decision rule, whereas the star corresponds to a threshold tuned on this curve; moving to it trades 4.2 points of specificity for 6.6 points of sensitivity.

*1D-CNN–LSTM Hybrid:* The design follows from an observation about the signal itself. Fatigue appears both as local change within a window and as a trend across windows, and a single mechanism would handle one of the changes better than the other. Two 1D convolutional layers (64 and 32 filters, kernel sizes 5 and 3) with batch normalization and max pooling therefore extract the local structure; a single 64-unit LSTM layer accumulates it over the sequence; a 32-unit dense layer and softmax produce the class probabilities (Figure 3). Formally,

(2) $${\hat{y}}_{t}=\mathrm{softmax}\left(W_{d}\;\cdot \;\mathrm{LSTM}\left(\mathrm{Conv}1D(x_{t-5:t})\right)+b_{d}\right)$$

where $x_{t-5:t}$ denotes the sequence of feature vectors spanning the current and five preceding analysis windows.

*CNN–BiLSTM–Attention:* The same convolutional front end feeding a bidirectional LSTM of 64 units per direction, followed by additive attention over the six output positions and a softmax head, as reported by Zhao et al. [55]. This is the most expressive model in the comparison and serves to test whether the accuracy of the hybrid is limited by its architecture or by the information available in the features.

### 4.3. Training Methodology

Partitioning is leave-operators-out: 16 operators for training, 4 for validation, and 4 held back for testing. The split is made at the operator level, never at the window level. This matters more than it might appear, since consecutive windows overlap by 50% and are strongly autocorrelated within a session; a random window-level split would place near-duplicates on both sides of the partition and inflate every reported figure.

The partition is then rotated over six disjoint test folds of four operators each. Training, validation-based early stopping, and threshold selection are repeated for every fold, so each operator is held out exactly once, and every prediction for a given operator comes from a model that was never trained on that operator’s data. The rotation is required by the design of the study; it was not adopted for rigor alone. Contribution C4, stated in Section 1.4, requires a fatigue estimate for all 24 operators, since the intervention arm of Section 6 reallocates tasks based on that estimate. Had a single fixed split been used, 20 of the 24 operators would either have gone unscored or been scored by a model trained on their own data, and neither outcome supports a valid claim. Every figure reported in Section 7 therefore comes from the pooled out-of-fold predictions, which cover all 24 operators and all 294,912 windows.

The 45:38:17 fatigue class ratio is handled by weighting the loss inversely to class frequency, $w_{k}\propto 1/p_{k}$, normalized so that $\sum_{k}w_{k}=3$. This gives weights of 0.62, 0.74, and 1.64 for fresh, moderate, and severe fatigue, respectively. Weighting was preferred to synthetic oversampling, because interpolating new minority-class examples in a 127-dimensional space whose components are physiologically coupled risks generating windows that no operator could produce [82].

Hyperparameters were selected by 5-fold cross-validation on the training partition, with folds again built at the operator level. Grid search sufficed for RF, SVM, and XGBoost; the five neural models were tuned by Bayesian optimization with early stopping on validation-balanced accuracy (patience 10 epochs), trained for at most 50 epochs at batch size 32.

### 4.4. Ablation and Sensitivity Protocols

Two controlled experiments accompany the main comparison, and both reuse the six-fold rotation without modification, so their results are directly comparable with the main comparison reported in Section 7.

The modality ablation retrains the 1D-CNN–LSTM hybrid from scratch on reduced feature vectors. Fifteen configurations are evaluated: the full 127-dimensional vector; five leave-one-block-out variants together with one that removes the inertial and biomechanical blocks jointly, since the latter is derived from the former; four single-block variants; three combinations of two or three blocks; and one reduced-instrumentation configuration corresponding to a plausible minimal deployment (two sEMG channels at the biceps brachii and anterior deltoid of the dominant side, two IMUs at the corresponding upper arm and forearm, and the three contextual variables, for 31 dimensions in total). Every configuration is retrained from scratch instead of being evaluated with features zeroed out, because zeroing leaves the network’s learned weights adapted to inputs that are no longer present and understates what a purpose-built reduced model would achieve. Hyperparameters are re-tuned within each configuration.

The threshold sensitivity analysis moves the FDI cut-point pair over five settings, from (30, 60) to (50, 80) in steps of 5, relabels the entire dataset accordingly, and repeats training and evaluation end-to-end. Class weights are recomputed for each setting, since the class balance changes with the thresholds. Three quantities are tracked: balanced accuracy, severe-class recall, and the mean lead time between the first alert and the crossing into the severe class.

### 4.5. Evaluation Metrics

The reported metrics are overall accuracy, balanced accuracy (macro-averaged recall), per-class precision and recall, macro-averaged F1, and one-versus-rest AUC [83]. Balanced accuracy is given alongside raw accuracy because the classes are unequal and the severe fatigue class—the one that matters operationally—is the smallest. Confidence intervals at 95% come from a cluster bootstrap with B=1000 resamples drawn over operators, not over windows. Resampling windows would treat 294,912 correlated observations as independent and return an interval several times too narrow; the operator is the unit of replication here, and there are 24 of them. Pairwise differences between architectures are tested on the 24 per-operator balanced accuracies with a Wilcoxon signed-rank test, the paired design being appropriate because every model is evaluated on the same operators [84].

Deployment cost is characterized by five quantities, since latency alone understates it: trainable parameter count, multiply–accumulate operations per inference expressed as floating-point operations (FLOPs), serialized model size in 32-bit floating-point format, peak resident memory during inference, and wall-clock latency. Latency, memory, and energy were measured on an NVIDIA Jetson Nano developer kit (NVIDIA Corporation, Santa Clara, CA, USA), a deliberately modest edge target: a quad-core Cortex-A57 at 1.43 GHz, a 128-core Maxwell GPU rated at 472 GFLOPS in half precision, 4 GB of LPDDR4 shared between CPU and GPU, and a 10 W power envelope [85]. The platform was chosen because it is roughly two orders of magnitude below a contemporary training GPU in arithmetic throughput and can be powered from the cell’s existing supply without additional cooling, so a model that meets its latency budget there will meet it on anything a plant is likely to install. Each figure is the median of 1000 single-sample inferences after 100 warm-up runs, with the board in its 10 W mode and the clocks pinned.

## 5. Biomechanical Fatigue Model

### 5.1. Musculoskeletal Model

Sensor readings become ergonomically meaningful only after they are mapped onto joint loads, and that mapping requires a musculoskeletal model. The one used here has 23 degrees of freedom (DOF) and is adapted from OpenSim (https://simtk.org/projects/opensim, accessed on 30 August 2026; National Center for Simulation in Rehabilitation Research, Stanford, CA, USA) [86]. The DOF count is made up as follows: seven DOF per upper limb, namely, three at the shoulder (flexion/extension, abduction/adduction, internal/external rotation), two at the elbow (flexion/extension, pronation/supination), and two at the wrist (flexion/extension, radial/ulnar deviation), taken bilaterally; three trunk DOF (flexion/extension, lateral bending, axial rotation); and six for the free-floating pelvis, which serves as the root segment. Segment lengths and inertial properties are scaled to each operator’s anthropometry by the procedure of Section 6.2 [87].

### 5.2. Inverse Dynamics and Load Estimation

Joint angles $q\left(t\right)\in R^{23}$ computed from IMU orientations via inverse kinematics optimization serve as inputs to Newton–Euler inverse dynamics computation:

(3) $$M\left(q\right)\ddot{q}+C\left(q,\dot{q}\right)\dot{q}+G\left(q\right)=\tau +J^{T}\left(q\right)F_{\mathrm{ext}}$$

where M(q) is the generalized mass matrix, $C(q,\dot{q})$ collects Coriolis and centrifugal terms, G(q) is gravitational loading, and the Jacobian transpose $J^{T}\left(q\right)$ projects the FSR-measured external force into joint space [88]. Equation (3) yields the net torques τ(t), which do not by themselves determine the individual muscle forces, since many combinations of muscle force produce the same net torque. The redundancy is resolved by static optimization over 92 muscle actuators [89]:

(4) $$\min_{f_{m}}\sum_{i=1}^{92}{\left(\frac{f_{m,i}}{f_{m,i}^{\max}}\right)}^{3}\;\mathrm{subject}\;\mathrm{to}\;R\left(q\right)f_{m}=\tau ,\;0\leq f_{m,i}\leq f_{m,i}^{\max}$$

with $f_{m,i}^{\max}$ the maximum isometric force of muscle *i* and R(q) the moment arm matrix. The cubic exponent is not arbitrary. It penalizes high activation disproportionately and, therefore, spreads load across synergists in a way that matches measured recruitment more closely than a linear or quadratic criterion would [90].

### 5.3. Personalized Fatigue Dynamics

Individual fatigue progression is modeled through an exponential force capacity decline:

(5) $$f_{\max}\left(t\right)=f_{\max}^{0}\;\cdot \;\exp\left(-\frac{t}{T_{i}}\right)$$

with $f_{\max}^{0}$ the initial maximum force capacity, *t* the cumulative time under load, and $T_{i}$ an operator-specific endurance time constant [91]. The symbol $T_{i}$ is used in place of the τ conventional in this literature, so as not to collide with the joint torque vector τ of Equation (3).

Identifying $T_{i}$ takes three maximum voluntary contraction trials to fix the 100% activation reference, followed by a sustained contraction at 50% MVC held to task failure. The whole protocol runs to about 30 min per operator. That is a real cost in production time, although it is short enough to be absorbed into an induction session or an annual occupational health check instead of being repeated on a working day. Across the 24 synthetic models, $T_{i}$ ranged from 85 s to 218 s, with a mean of 142±48 s—a coefficient of variation of 34%. A spread of that size is the argument for identifying $T_{i}$ for each operator individually; a population mean would serve no one well. Differences in time to task failure between men and women are also not universal across tasks: comparable endurance has been reported for bilateral isometric leg extension [92], which cautions against treating sex as a substitute for individual measurement.

### 5.4. Integration with Sensor Data

The biomechanical model and the sensor stream are coupled in both directions. In one direction, IMU kinematics and FSR forces enter the inverse dynamics computation of Equation (3) as inputs. In the other, the joint torques and muscle forces that computation returns are converted into twelve features and rejoin the remaining 115: peak shoulder torque, peak elbow torque, cumulative shoulder load, cumulative elbow load, peak grip force, and mechanical work, each computed bilaterally to make up the 127-dimensional vector.

Equation (5) also supplies the training fatigue labels, through the FDI defined in Section 4.1. Taking the ground truth from simulation state instead of self-report removes the annotation ambiguity that affects human fatigue studies, in which a reported score reflects motivation, personality, and social context as well as physiology. The price is that the labels are only as good as the model producing them. Section 7.7 and Section 8.4 quantify and delimit that dependency, respectively.

## 6. Digital Twin Validation Framework

### 6.1. Relationship Between the Instrument and the Twin

Because two distinct artifacts appear in this paper, it is useful to set out how they relate, extending the general description of human digital twins given in Section 2.4. The wearable platform of Section 3 is a specification of physical instrumentation: particular sensors, at particular anatomical sites, with particular sampling rates, noise floors, and radio characteristics. The digital twin described in this section is a three-stage simulation that (i) drives a 23-DOF musculoskeletal model through an assembly task, (ii) computes the internal fatigue state of that model exactly, and (iii) synthesizes the signals that the specified instrumentation would have produced had it been worn by an operator in that state.

The twin therefore plays two roles at once: it is the source of labeled training data, and it is the test bench on which the acquisition, conditioning, feature extraction, and inference chain is exercised end-to-end before any hardware is procured. In a deployment with human operators, the hardware of Section 3 would replace stage (iii): real sEMG, inertial, and force streams would enter the same conditioning and feature pipeline, and the trained model would run unchanged. Stages (i) and (ii) would remain in place, since inverse dynamics still requires a scaled musculoskeletal model to convert measured kinematics into joint torques, and 12 of the 127 features come from that computation. What would disappear is the exact FDI label, which is available only inside the simulation; a deployed system would either run open-loop on the trained classifier or be re-anchored against a periodic MVC test.

This raises the question of which model a given operator would be paired with. Two arrangements are possible, and they are not equivalent. Matching the operator to the nearest existing synthetic model by anthropometry is cheap and requires no measurement beyond height, mass, and segment lengths, but it inherits whatever endurance time constant that model was assigned, and Section 5.3 shows that the constant varies by a factor of 2.6 across the cohort. Building a model for the operator, by scaling the generic musculoskeletal model to measured anthropometry and identifying $T_{i}$ through the 30 min protocol, is the arrangement assumed throughout this paper, and Section 8.3 quantifies how much accuracy is at stake in that choice. The practical recommendation that follows is a hybrid: anthropometric scaling on the first day, so that the system is usable immediately, with $T_{i}$ identification scheduled into the first occupational health appointment.

### 6.2. Synthetic Operator Model Library

Twenty-four synthetic operators were generated to span the anthropometric range of the U.S. manufacturing workforce. Generation proceeded in four steps:

First, the joint distribution of stature, body mass, and eight segment lengths was taken from the ANSUR-II survey [93], stratified by sex, and the age distribution from Bureau of Labor Statistics manufacturing employment data [94]. Second, 24 parameter vectors were drawn from that joint distribution by Latin hypercube sampling rather than by simple random draw, so that the cohort covered the marginal ranges evenly at a sample size too small for random sampling to do so reliably; the design was constrained to 12 male and 12 female models. Third, each parameter vector was used to scale the generic 23-DOF musculoskeletal model of Section 5, with segment inertial properties following standard regression equations on stature and mass, and maximum isometric muscle forces scaled by physiological cross-sectional area [87]. Fourth, an endurance time constant $T_{i}$ was assigned to each model by drawing from a log-normal distribution fitted to published occupational endurance data [91], with group means of 156±51 s and 128±42 s for male and female models, respectively, following reported differences in fatigability [11]; the draw was independent of the anthropometric parameters, since the empirical association between the two is weak.

Table 3 summarizes the resulting cohort. Kolmogorov–Smirnov tests against the reference distributions, reported in Table 3, do not distinguish the sampled cohort from the target population in terms of stature, mass, or body mass index. What the procedure does not reproduce is any correlation between anthropometry and work technique, since every model executes the same task-level primitives.

### 6.3. Simulation Environment

The collaborative work cell was built in CoppeliaSim v4.5 (Coppelia Robotics AG, Zurich, Switzerland) [95]: three UR5e manipulators (Universal Robots A/S, Odense, Denmark), positioned at 120° intervals around a central workstation, inside a 6×4×3 m envelope. Robot kinematic and dynamic parameters are the manufacturer’s; collision geometries are retained so that ISO/TS 15066 separation monitoring can be reproduced. The digital humans are driven through Lua scripts acting on the 23-DOF chain, with motion generated from task-level primitives retargeted from the CMU Graphics Lab Motion Capture Database [96].

An assembly cycle comprises part retrieval (components of 0.5–3 kg at reach distances of 40–80 cm), installation of 12 fasteners at 20 Nm, inspection, and packaging. Variability enters through eight randomized part orientations and eight bin positions, so that no two cycles impose an identical loading pattern. Forward dynamics runs on Bullet Physics v2.83 at 200 Hz, with contact parameters set for metal-on-metal contact.

### 6.4. Synthetic Sensor Data Generation

Signals for all three measurement modalities are synthesized to match the specifications of the target hardware listed in Table 1, so the pipeline can be exercised end-to-end before any of it is built.

sEMG signals are generated through a three-stage pipeline: (1) neural drive computation via static optimization yielding activation levels $a_{i}\left(t\right)\in \left[0,1\right]$ for 92 muscles, (2) excitation–contraction coupling through first-order dynamics (activation time constant 30 ms), and (3) surface potential generation:

(6) $${\mathrm{EMG}}_{i}\left(t\right)=\sqrt{a_{i}\left(t\right)}\;\cdot \;G_{i}\;\cdot \;N\left(0,\sigma^{2}\right)+n\left(t\right)$$

where $G_{i}$ is a muscle-specific gain in the 0.8–1.2 mV range, $N(0,\sigma^{2})$ supplies amplitude modulation with σ=0.15, and n(t) is additive noise set to give an SNR of 24 dB [97]. The square-root dependence on activation reproduces the empirical amplitude–force relationship, which is not linear. Spectral compression is imposed on top of this by shifting the pole frequencies of the shaping filter in proportion to the instantaneous force capacity of Equation (5), which is the mechanism that makes the synthetic median frequency fall as the simulated operator fatigues. IMU signals come from forward kinematics with Gaussian noise matched to Xsens MTw specifications ($\sigma_{a}=0.01\;ms^{-2}$, $\sigma_{\omega }=0.1{}^{\circ}\;s^{-1}$) [61]; FSR signals come from contact dynamics, with the piezoresistive nonlinearity reproduced and 0.5 N of measurement noise added [63].

Two further degradations were injected deliberately, since a pipeline tested only on clean data will fail on its first work shift. Packets were dropped stochastically at 1.7% for sEMG, 0.9% for IMU, and 2.2% for FSR, and wireless latency was drawn from an exponential distribution with mean 50 ms.

### 6.5. Experimental Protocol

The experimental design mirrors a within-operator crossover trial. Every synthetic operator completes 8 sessions—4 baseline and 4 intervention—spread over 4 virtual weeks with a 48 h washout between them; order is assigned by Latin square, giving 24×8=192 sessions. Each session runs for 8 h with breaks of 15 min at 2 h, 30 min at 4 h, and 15 min at 6 h.

In baseline sessions, the operator follows a fixed task sequence and responds to fatigue only reactively, with movement velocity being scaled by 0.7+0.3(1−f), with f∈[0,1] the current fatigue level. No reallocation occurs in this condition, whatever the loading.

In intervention sessions, the prediction output is allowed to act through a trigger with two tiers that fire on different quantities and at different times.

*Predictive Tier:* The classifier is evaluated once every 15 s on the 30 s window ending at that instant, and it returns a posterior over the three fatigue states. Reallocation is requested when the posterior probability of the severe state exceeds 0.70. This is the only tier that can act before the loading that it is meant to prevent has occurred, and it is therefore the tier responsible for the lead time reported in Section 7.8: the classifier responds to the trajectory of the descriptor—in particular, to the joint drift of median frequency, RMS amplitude, and cumulative shoulder moment across the six windows in its input—rather than to any level having been reached. Section 7.6 reports what happens to the lead time when the class cut-points that define the severe state are moved.

*Reactive Tier:* Two fixed biomechanical thresholds are evaluated on the same cadence, from the joint moments that the twin computes by inverse dynamics: 70% MVC sustained for more than 45 s, or a cumulative shoulder moment exceeding 15 Nm·h. These are backstops. They fire on exposure that has already been sustained, so they cannot anticipate anything, and the first of them coincides with the definition of an injury-risk event used in Section 7.8, which means that it can shorten such an event but not prevent one. The second threshold has no direct counterpart in published exposure limits, which address lifting and hand activity but not accumulated shoulder moment [98]; it was adopted by analogy with the cumulative-exposure logic that those limits embody, and it should be read as a working value with no standing as a standard.

A second, lower level at a predicted probability of 0.50 separates the fresh and moderate zones of the monitoring traces reported in Section 7.8. It is displayed to the operator as a warning and does not by itself request reallocation, so 0.70 remains the only probability at which the allocator acts.

A request from either tier is passed to the cell task allocator, which selects among three strategies: redistributing subtasks to the collaborative robot, which removes roughly 40% of shoulder loading; inserting a 2 min active microbreak every 30 min while risk remains elevated; and adjusting bin height, which reduces shoulder flexion by about 25°. Robot reallocation is the strategy that determines the outcome reported in Section 7.9: the 43% reduction in peak shoulder moment is close to the 40% that strategy removes by construction, and the 6% throughput cost follows from the robot performing the reallocated subtask more slowly than the operator that it relieves.

## 7. Experimental Results

All results in this section were obtained in the simulated environment described in Section 6. They characterize the behavior of the pipeline on synthetic signals whose statistical properties were specified by the authors, and Section 8.4 states what may and may not be inferred from them about a physical deployment.

### 7.1. Model Performance Comparison

Table 4 reports the pooled out-of-fold results over all 24 operators and 294,912 windows; Figure 3 shows the same comparison graphically.

The CNN–BiLSTM–attention model gives the highest raw accuracy, at 89.6%, ahead of the 1D-CNN–LSTM hybrid by 0.3 percentage points. That margin is not statistically meaningful: the confidence intervals overlap almost completely, and a Wilcoxon signed-rank test on the 24 per-operator balanced accuracies returns p=0.61. The same holds for the Transformer encoder (p=0.47) and for the plain LSTM (p=0.19). What separates the eight models reliably is not the choice among sequential architectures but the presence or absence of temporal context, and this is what Table 4 shows most clearly. Every sequential model lies above every instantaneous one, the gap between the two groups runs from 1.5 to 7.5 accuracy points, and each difference across the group boundary is significant at p<0.01, whereas none within the sequential group is. Temporal context therefore buys more than architectural sophistication does, a finding that concerns the structure of the problem more than any particular network.

Two observations about the advanced baselines should be recorded, since they were introduced specifically to test whether the hybrid was leaving accuracy unclaimed. The Transformer encoder does not benefit from its capacity at this sequence length. Six positions give self-attention very little to attend to, and the learned attention weights were close to uniform in every fold, so the model reduces in practice to a mean-pooled feed-forward network over convolution-free inputs. The TCN, by contrast, matches the recurrent models to within 0.3 points of accuracy while remaining fully parallel over the sequence, and Section 7.2 shows what that buys in latency. Every model stays under 40 ms on the Jetson Nano, so inference consumes less than half of what a controller can tolerate; Section 3.2 accounts for the remainder of the end-to-end path and for the interval within which it falls.

### 7.2. Model Complexity and Deployment Cost

**Figure 4:**
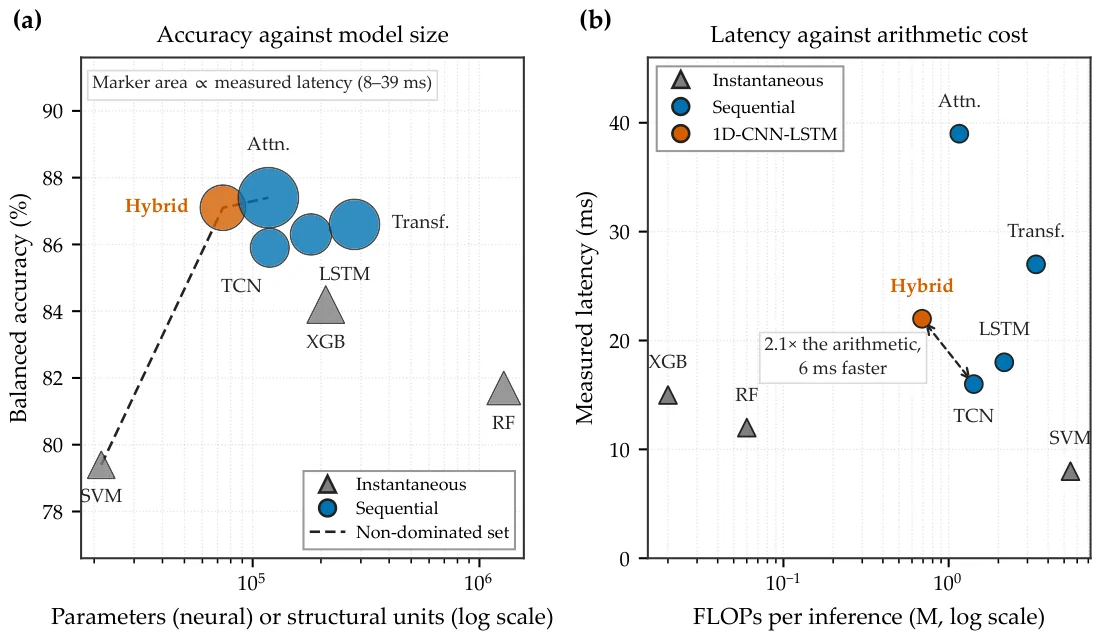
Accuracy against computational cost for the eight architectures. (**a**) Balanced accuracy versus trainable parameter count (logarithmic axis), with marker area proportional to measured Jetson Nano latency; the dashed contour joins the models that are not dominated—that is, those for which no other model is simultaneously more accurate and cheaper. (**b**) Measured latency against analytically counted FLOPs, showing that the two orderings do not coincide: recurrent models pay a sequential-execution penalty that arithmetic cost does not capture, whereas the temporal convolutional network executes its larger operation count in parallel.

Latency alone understates what a model costs to deploy. Table 5 adds parameter count, arithmetic cost, serialized size, peak memory, and measured energy, all obtained on the Jetson Nano configuration described in Section 4. Figure 4 plots balanced accuracy against parameter count, with latency encoded in marker size.

The hybrid is the smallest neural model in the comparison, at 73.9 k parameters and 0.28 MB, roughly one quarter the size of the Transformer encoder and 38% smaller than the attention model that edges it on accuracy. Its arithmetic cost is also the lowest of the five sequential architectures, at 0.69 MFLOPs per inference. The CNN–BiLSTM–attention model buys 0.3 accuracy points at 1.6 times the parameters, 1.7 times the FLOPs, and 1.8 times the latency. That comparison is the clearest single argument for the hybrid, and it is an argument about cost rather than about accuracy.

Latency and FLOPs do not rank the models identically, which Figure 4b makes explicit. The TCN performs twice the arithmetic of the hybrid and still runs 6 ms faster, because its dilated convolutions are evaluated in parallel across the sequence, whereas an LSTM must advance through its six timesteps in order. On this platform, the per-timestep kernel launch overhead dominates the multiply–accumulate cost, so a model that halves its recurrent depth gains more than one that halves its operation count. Anyone selecting an architecture from published FLOPs counts alone would reach the wrong conclusion here.

Three quantities bear directly on deployment feasibility. Peak resident memory never exceeds 118 MB, so all eight models fit comfortably in the 4 GB the board provides, and the gateway could host the inference process alongside the conditioning stage without contention. Energy per inference is at most 148 mJ; at the one-inference-per-15 s cadence that the feature extractor imposes, this corresponds to a mean incremental power of 9.9 mW, negligible against the board’s idle draw and immaterial for a mains-powered cell. Finally, post-training INT8 quantization of the hybrid [39] reduced the serialized model to 0.09 MB and latency to 14 ms at a cost of 0.3 points of balanced accuracy, which brings the end-to-end budget of Section 3.2 down to 79–119 ms.

### 7.3. Confusion Matrix and Per-Class Analysis

**Figure 5:**
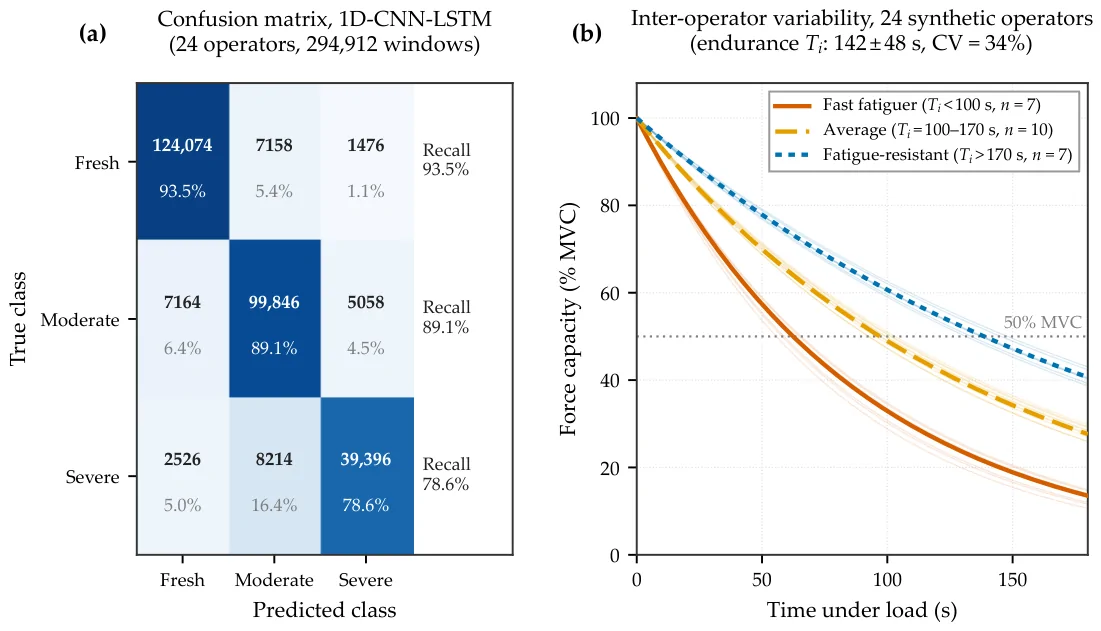
(**a**) Three-class confusion matrix for the 1D-CNN–LSTM hybrid model on the pooled out-of-fold predictions. Cells give window counts; rows sum to 132,708 fresh, 112,068 moderate, and 50,136 severe fatigue windows, for 294,912 in total. Cell shading encodes the row-normalized proportion on a single-hue blue scale, from white at 0% to full saturation at 100%, so color duplicates the printed value and carries no independent information. (**b**) Inter-operator variability in force capacity decline across 24 synthetic operator models grouped into three phenotypic clusters by the endurance time constant $T_{i}$ of Equation (5), illustrating the 34% coefficient of variation that motivates per-operator calibration. Each cluster is identified by both color and line style—fast fatiguers in orange with a solid line, average operators in yellow with a dashed line, and fatigue-resistant operators in blue with a dotted line—so the panel is readable in greyscale; faint lines of the same color are the individual operators contributing to each cluster mean, and the horizontal dotted line marks 50% of maximum voluntary contraction. The symbol $T_{i}$ is used in place of the conventional τ in this literature, which Equation (3) reserves for the joint torque vector.

Figure 5 presents the confusion matrix for the 1D-CNN–LSTM hybrid and inter-operator variability across operator phenotypes.

Table 6 gives the per-class figures that the matrix implies.

The three classes are not recognized with equal reliability. Fresh is identified most reliably, at 93.5% recall for 92.8% precision, and its signature of high median frequency together with low RMS amplitude makes that unsurprising. Moderate follows, at 89.1% recall and 86.7% precision, which is what a transitional state between two well-separated endpoints should give. Severe fatigue reaches 85.8% precision but only 78.6% recall. Averaging the three F1 values unweighted gives the 87.7% reported in Table 4.

The asymmetry in the severe fatigue class runs in the unfavorable direction, and that needs saying. Precision of 85.8% means few false alarms, which protects operator trust in the system. Recall of 78.6% means that roughly one severe episode in five is read as moderate. This is the error that a safety system can least afford, and it follows in part from setting the class weights by class frequency alone. Weighting the severe fatigue class more aggressively than $1/p_{k}$ would trade precision for recall; for a deployed system, that trade-off is probably worth making.

Grouping the 24 operators by endurance time constant gives three clusters: fast fatiguers ($T_{i}<100\;s$, n=7), average ($T_{i}=100$–170 s, n=10), and fatigue-resistant ($T_{i}>170\;s$, n=7). Accuracy tracks the grouping, from 85.1% for the fast fatiguers to 92.8% for the resistant ones. The ordering is the opposite of what a safety engineer would want, since the operators at greatest risk are the ones that the classifier handles worst.

### 7.4. Modality Ablation

**Figure 6:**
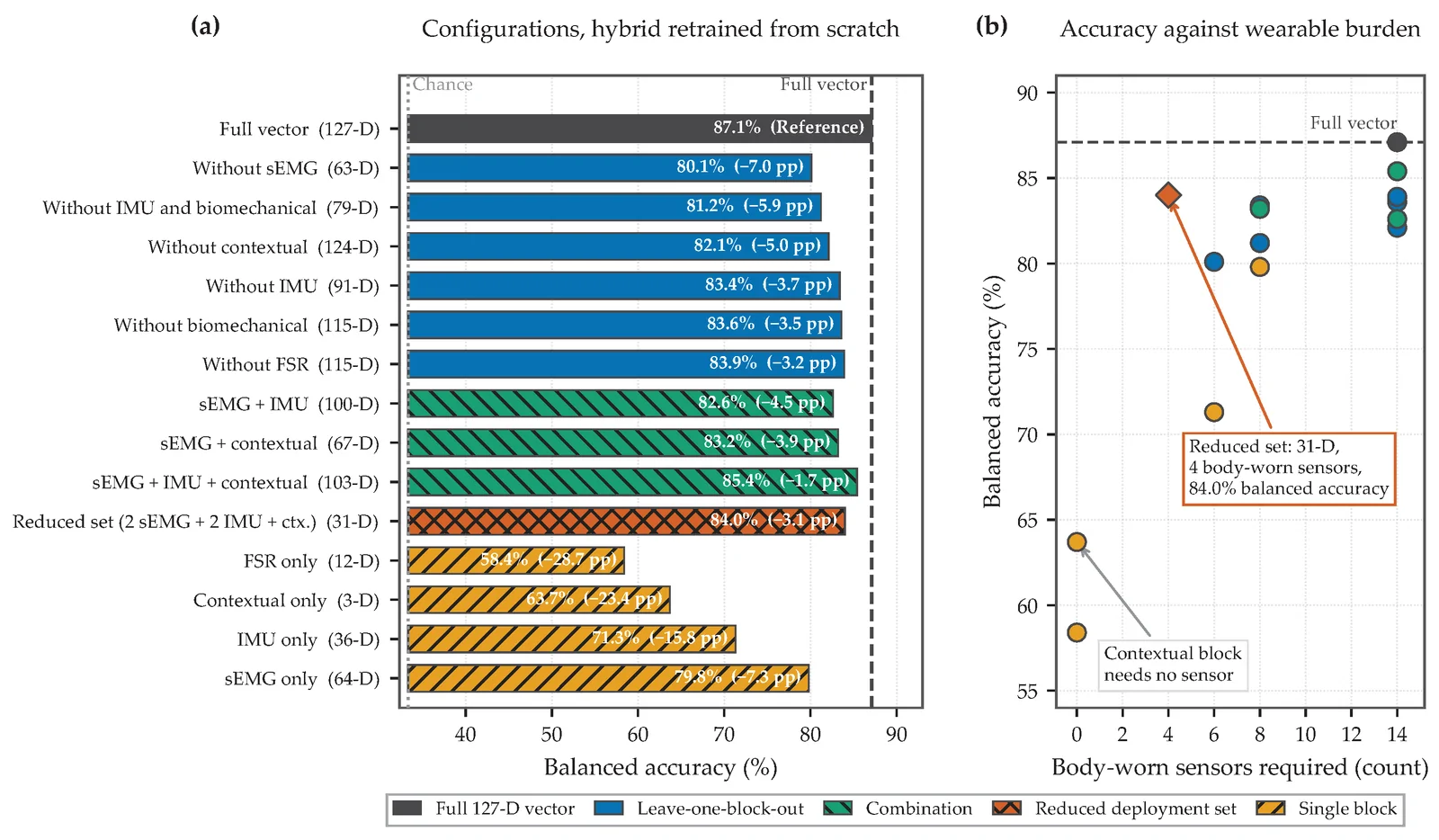
Contribution of each sensing modality to fatigue classification. (**a**) Balanced accuracy for the leave-one-block-out and single-block configurations of Table 7, with the full-vector result marked by a horizontal line and the three-class chance level at 33.3% marked by a second. (**b**) Balanced accuracy against the number of body-worn sensors each configuration requires, with the reduced 31-dimensional set highlighted. Marker shape encodes configuration family: the diamond is the reduced deployment set, the black circle the full 127-dimensional vector, and the remaining circles the leave-one-block-out, combination, and single-block configurations, colored as in the legend of panel (**a**). The horizontal dashed line is the full-vector reference, and the two leader lines connect the annotation boxes to the points they describe. The contextual block requires no body-worn sensor, which places the configurations that use it alone on the vertical axis.

Table 7 reports the fifteen configurations specified in Section 4.4, each a full retraining of the hybrid under the same six-fold rotation. Figure 6 plots the same results against the number of instrumented channels that each configuration requires.

Removing sEMG costs 7.0 points of balanced accuracy, more than any other block, which is the ordering one would expect, since sEMG observes neuromuscular activation directly. The second-largest decrement, 5.0 points, comes from the three contextual variables, which require no sensor at all. That two clock readings and a cycle counter should outweigh the entire six-unit inertial array is the most consequential result in the table, and Section 8.1 takes up what it implies for deployment.

The single-block rows show that no modality is sufficient alone. sEMG on its own reaches 79.8%, only 0.3 points above the configuration that omits it entirely—a signature of substantial redundancy: the information that sEMG carries is largely, though not entirely, recoverable from the other four blocks combined. The combinations quantify that redundancy directly. sEMG alone gives 79.8% and IMU alone 71.3%, yet the two together reach only 82.6%, well short of what independence would predict. Adding the contextual block to the same pair lifts the result by a further 2.8 points to 85.4%, the largest marginal gain per added dimension anywhere in the table, because elapsed exposure is the one quantity that no instantaneous physiological measurement encodes.

The reduced 31-dimensional configuration is included because it corresponds to instrumentation that a plant might actually accept: two electrodes and two inertial units on the dominant arm, plus data that the manufacturing execution system already holds. It retains 84.0% balanced accuracy, 3.1 points below the full platform, with 4 body-worn sensors instead of 14. Section 8.7 returns to this configuration.

### 7.5. Feature Importance Analysis

**Figure 7:**
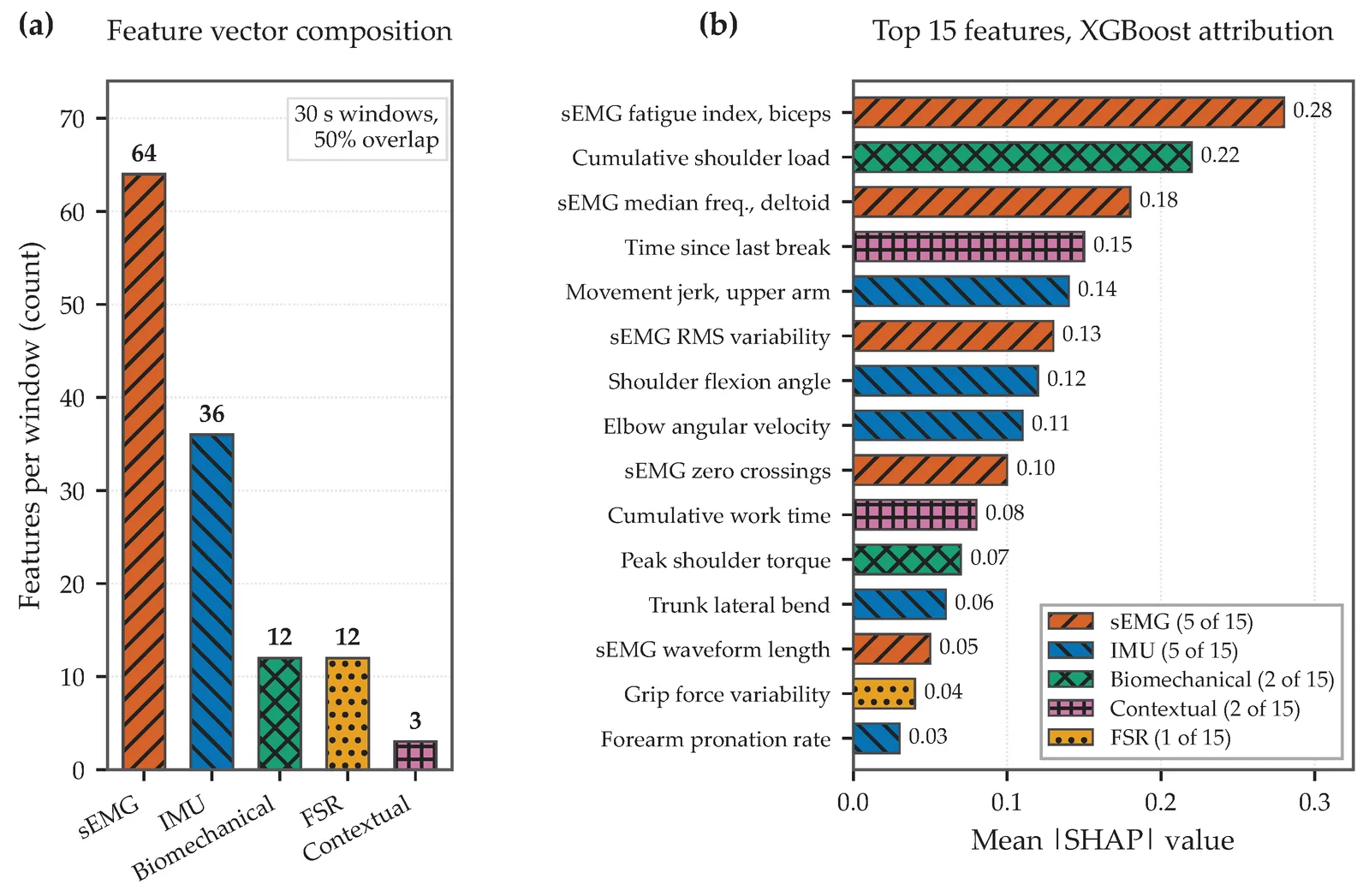
Feature engineering composition and SHAP-based importance analysis. (**a**) Breakdown of the 127-dimensional feature vector by sensor modality, showing the dominant contribution of sEMG channels (64 features) followed by IMU kinematics (36 features), with biomechanical, FSR, and contextual features completing the representation. (**b**) Top 15 features ranked by mean absolute SHAP value computed from the XGBoost model, color-coded by modality: sEMG features dominate the ranking, with cumulative shoulder load—a biomechanical feature—and elapsed time since the last break—a contextual feature—providing complementary discriminative power.

SHAP attributions were computed with the SHAP package (https://shap.readthedocs.io, accessed on 30 August 2026) on the XGBoost model, whose tree structure admits exact Shapley values at tractable cost; the hybrid would require sampling approximations. Figure 7 ranks the top 15 features by mean absolute SHAP value.

sEMG dominates the head of the ranking. The biceps brachii fatigue index comes first (mean |SHAP| = 0.28) and anterior deltoid median frequency third (0.18), in the order that the task structure would predict: the biceps and deltoid are the prime movers for the reaching and lifting that occupy most of an assembly cycle. Cumulative shoulder load takes second place (0.22), so accumulated mechanical stress carries information that the localized neuromuscular signals do not.

Elapsed time since the last break comes fourth (0.15), and cumulative work time tenth (0.08). Neither requires a sensor. That two clock readings should outrank most of the instrumented channels is consistent with the ablation result of Section 7.4, and the two analyses agree despite resting on different models and different logic: one attributional and one based on retraining.

Across the top 15, the split is five sEMG, five IMU, two biomechanical, two contextual, and one FSR. No modality has the field to itself.

### 7.6. Sensitivity to the Fatigue Class Thresholds

**Figure 8:**
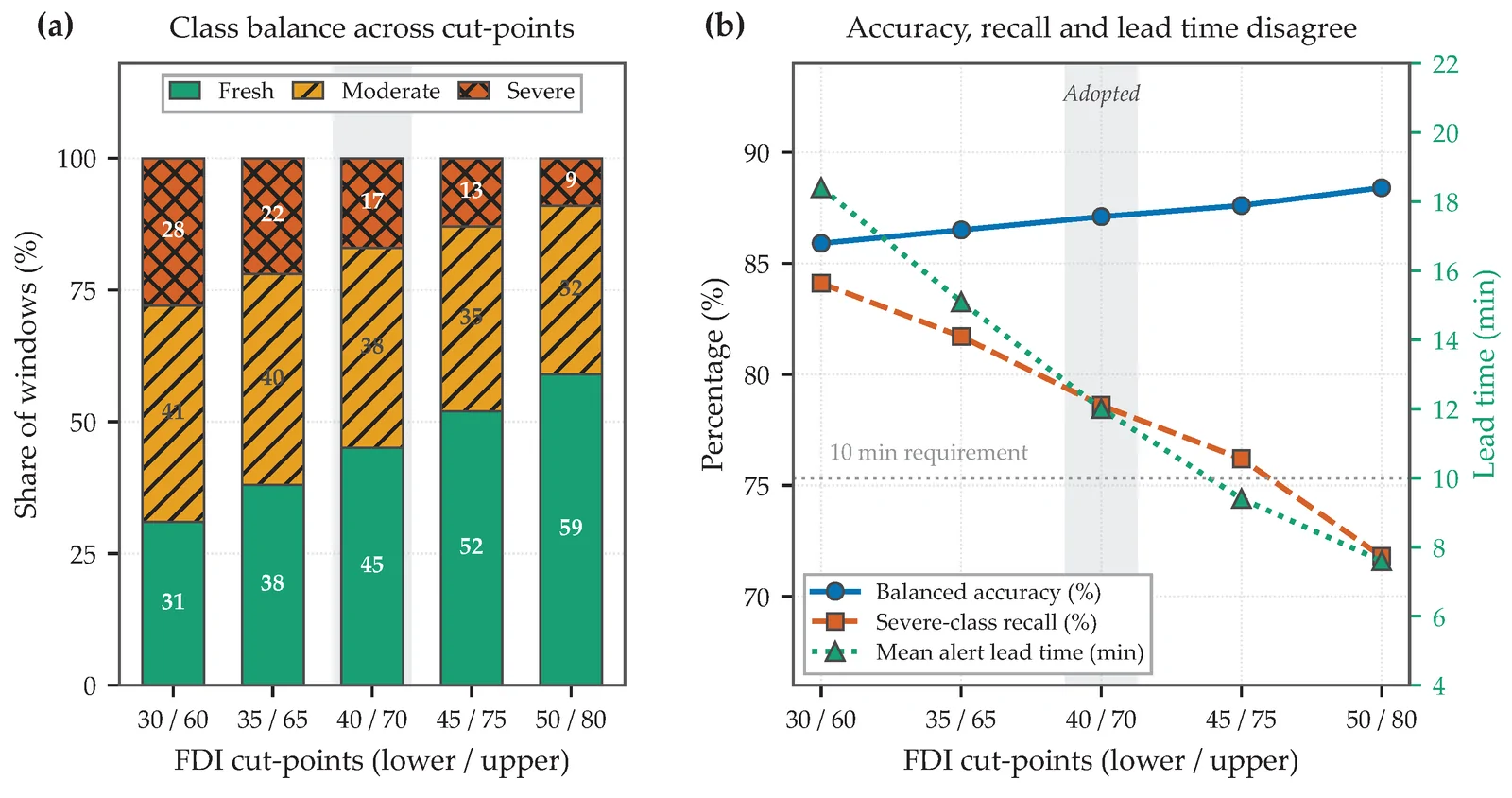
Effect of the fatigue class cut-points on classification and on alerting. (**a**) Class balance across the five cut-point settings, showing how raising the thresholds shrinks the severe fatigue class. (**b**) Balanced accuracy, severe-class recall, and mean alert lead time as functions of the cut-point pair; the vertical band marks the (40, 70) setting adopted in the rest of the paper. Balanced accuracy rises monotonically with the thresholds, while severe-class recall and lead time fall, so the three curves do not share an optimum.

Table 8 and Figure 8 report what happens when the FDI cut-point pair is moved across the range specified in Section 4.4.

Classification performance is stable in the sense that matters: balanced accuracy varies by 2.5 points across a range of cut-points spanning 20 FDI units, and the ranking of the eight architectures is unchanged at every setting. What is not stable is the operational meaning of the classifier. Balanced accuracy improves monotonically as the thresholds rise, from 85.9% at (30, 60) to 88.4% at (50, 80), for a reason that has nothing to do with better discrimination: a higher cut-point moves the severe fatigue boundary further from the bulk of the data, where the classes overlap least. Over the same range, severe-class recall falls by 12.3 points, and mean alert lead time falls from 18.4 min to 7.6 min.

The three curves therefore have no common optimum, and reporting balanced accuracy alone would recommend the setting with the worst safety behavior. The (40, 70) pair was retained because it is the highest setting at which the mean lead time still exceeds 10 min, the interval that the intervention strategies of Section 6 require to reallocate a subtask or schedule a microbreak. A facility whose interventions are faster could raise the thresholds and gain accuracy; one relying on work shift rescheduling would have to lower them and accept the loss.

### 7.7. Construct Validity of the Simulated Fatigue Labels

Because FDI is defined inside the simulation, it is reasonable to ask whether it behaves like the fatigue indicators that would be measured from a human operator. Table 9 reports Spearman rank correlations between FDI and seven sensor-derived quantities, computed within each operator and pooled across the cohort by Fisher *z* transformation.

The pattern reproduces the canonical electrophysiological signature: median frequency falls and RMS amplitude rises as FDI increases, and the composite fatigue index of Equation (1), which combines both, tracks FDI most closely of all. The magnitudes are also in the range that published human studies report. Median frequency declined at a mean rate of 0.44% per minute across the cohort, with a per-operator range of 0.19 to 0.79% per minute, against the 0.3 to 0.8% per minute typically reported for sustained submaximal upper-limb effort [6,67]. Simulated force capacity fell by 19.6% per hour on average (range 11.2–28.7%), inside the 15–25% band reported for comparable assembly-type work [3]. Movement smoothness and grip variability correlate more weakly, which is also what the human literature would predict, since both are behavioral consequences of fatigue mediated by compensation strategy, which places them a step removed from the underlying state.

One qualification is essential. The FDI and the sEMG descriptors against which it is correlated are both outputs of the same generative model, and the spectral compression mechanism of Section 6.4 was written into that model deliberately. The correlations in Table 9 therefore establish internal consistency: they confirm that the synthetic signals carry the fatigue signature at physiologically plausible magnitudes, and that the classifier is not exploiting some artifact of the generator unrelated to fatigue. They are not external validation, they do not test whether the underlying fatigue model is correct, and no claim about human physiology follows from them. Only a study with human participants can supply that, and Section 8.4 states what such a study would need to measure.

### 7.8. Real-Time Prediction Performance

**Figure 9:**
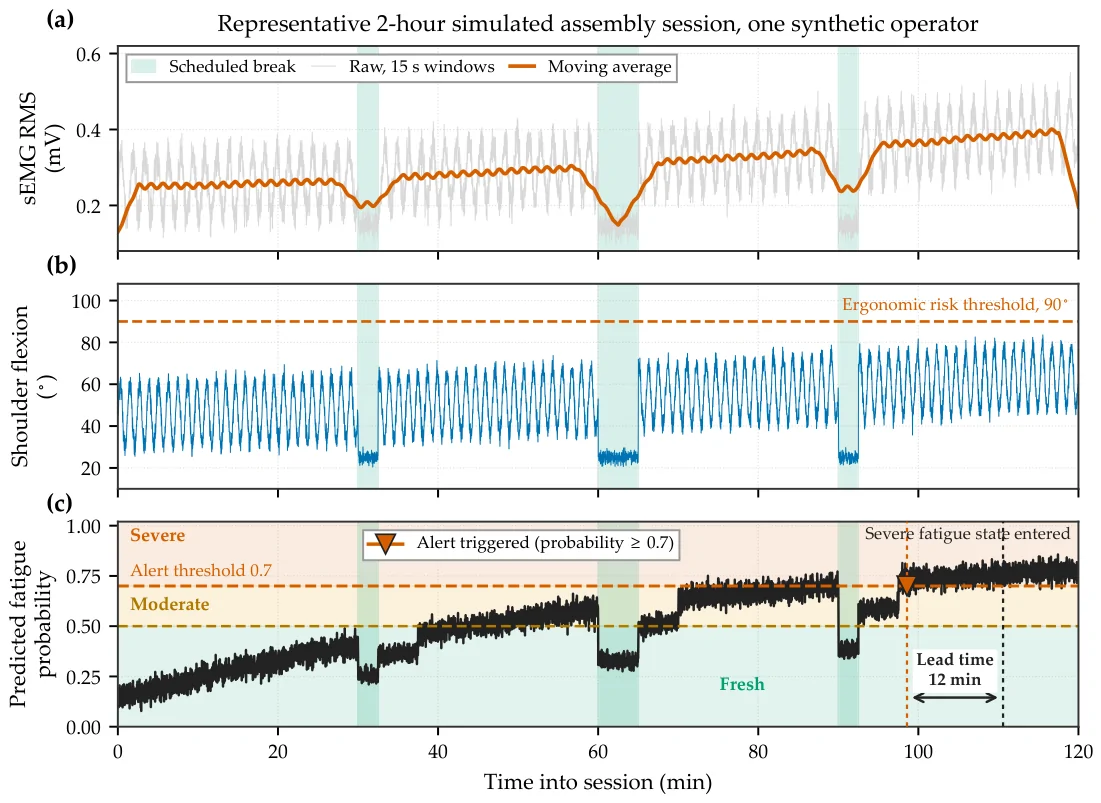
Real-time fatigue monitoring during a representative 2 h simulated assembly session for a single synthetic operator. The three panels share a common time axis, and the vertical green bands mark the three scheduled rest breaks. (**a**) sEMG RMS amplitude from the biceps brachii channel: the thin grey trace is the per-window value at the 15 s stride and the thick orange trace its moving average, showing progressive activation increase punctuated by partial recovery during the breaks. (**b**) IMU-derived shoulder flexion angle, plotted as a single blue trace, with the 90° ergonomic risk threshold drawn as a horizontal orange dashed line. (**c**) Model-predicted probability of the severe fatigue state, drawn as a black trace over three shaded zones that correspond to the predicted class—green for fresh below 0.50, amber for moderate between 0.50 and 0.70, and pale orange for severe above 0.70. The orange dashed line is the alert threshold at 0.70 and the amber dashed line the warning level at 0.50; the inverted orange triangle marks the instant at which the alert fires, the vertical black dotted line the instant at which the operator enters the severe state, and the double-headed arrow between them the lead time, which averages 12 min across the cohort. The line at 0.5 separates the fresh and moderate zones and is displayed as a warning only; reallocation is requested at 0.7, as specified in Section 6.5.

Figure 9 follows the system through a representative 2 h session, showing how the physiological signals, the model output, and the alert logic relate in time.

Alerts precede the crossing into the severe fatigue state by 12 min on average. How useful that margin is depends entirely on the intervention that it triggers. It is ample for reallocating a subtask or inserting a microbreak, and irrelevant to anything requiring an immediate stop. Across all 192 sessions, the system reached 87% sensitivity and 91% specificity for elevated injury-risk events, defined as joint torque above 70% of individual maximum capacity sustained beyond 45 s.

### 7.9. Intervention Effectiveness

**Figure 10:**
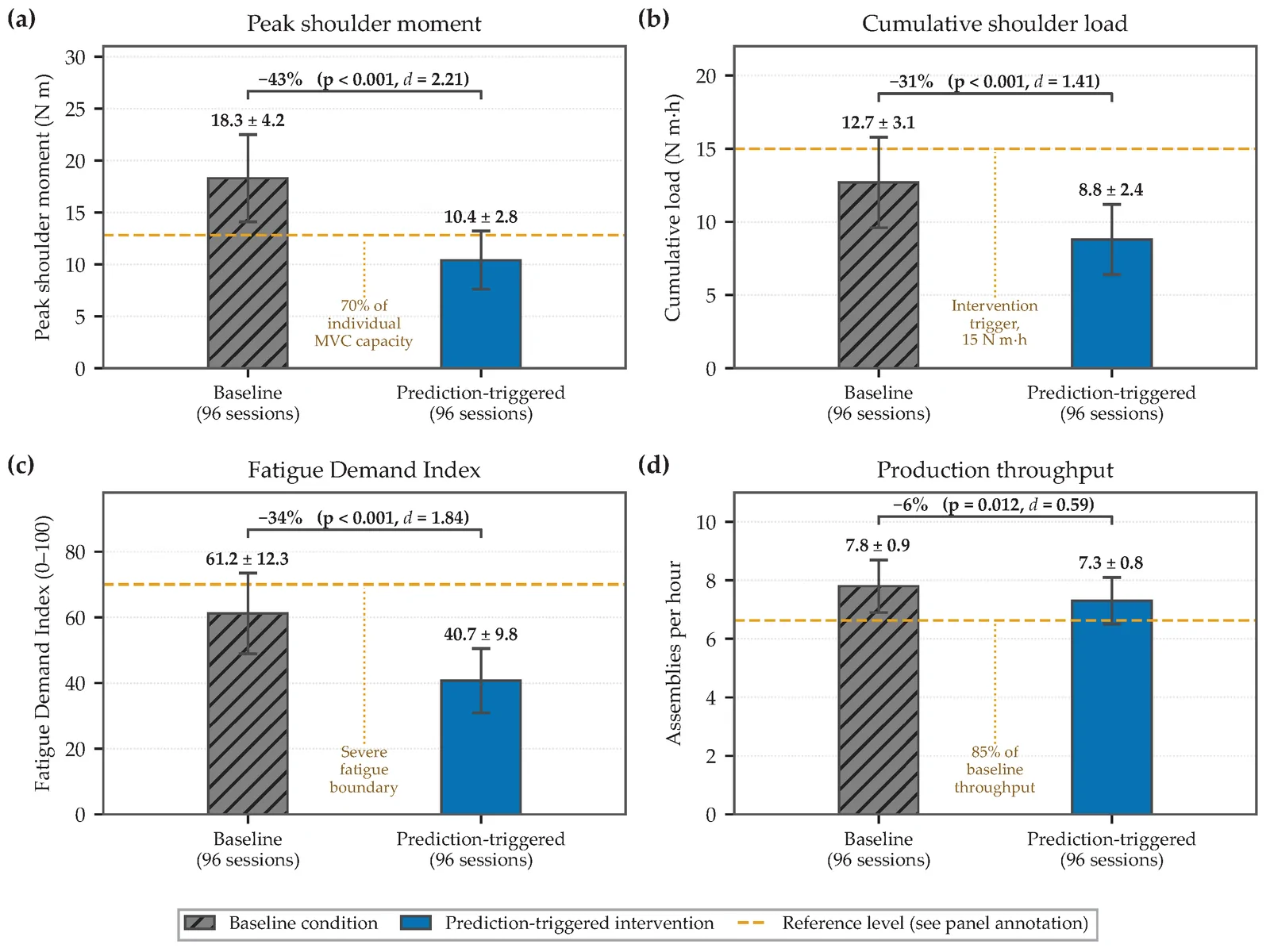
Intervention effectiveness evaluated across 192 simulated sessions (96 baseline, 96 prediction-triggered) using a within-operator crossover design. (**a**) Peak shoulder moment reduced by 43% (p<0.001, Cohen’s d=2.21) from baseline to intervention condition. (**b**) Cumulative musculoskeletal load, reduced by 31%; the dashed line marks the 15 Nm·h cumulative shoulder moment threshold adopted as the intervention trigger. (**c**) Fatigue Demand Index reduced by 34%, bringing the cohort mean from the middle of the moderate band to the boundary of the fresh zone. (**d**) Production throughput at 94% of baseline (p=0.012). Error bars indicate ±1 SD across operators.

Figure 10 and Table 10 give the effect of alert-triggered intervention on loading and on throughput.

Peak shoulder moment falls by 43%, from 18.3±4.2 to 10.4±2.8 Nm (p<0.001, Cohen’s d=2.21); cumulative loading by 31%; injury-risk events per work shift by 88%. Throughput drops 6%, from 7.8±0.9 to 7.3±0.8 assemblies per hour (p=0.012). Whether that trade-off is acceptable is a question of plant economics rather than of measurement, and it is taken up in Section 8.6.

These effects are produced by a classifier and a set of fixed thresholds acting together, and the two contributions should not be conflated. Separating them numerically would require a third arm in which the reactive tier drives reallocation with the classifier disabled, and that arm was not run. Two parts of the effect can still be assigned from the structure of the trigger. The anticipation belongs entirely to the classifier: the reactive thresholds fire only once the exposure that they define has been sustained, so a threshold-only policy has a lead time of zero by construction, and the 12 min in Section 7.8 is not available to it. The 88% reduction in injury-risk events belongs almost entirely to the classifier for the same reason, since an injury-risk event is defined by the same condition as the first reactive threshold: a threshold-only policy can truncate such an event but cannot prevent one from being counted. The reductions in peak shoulder moment and in cumulative load are the joint effect, because their magnitude is fixed by the strategies and by where the two thresholds are set, not by the accuracy of the prediction; a classifier of any accuracy that triggered the same robot reallocation would remove the same 40% of shoulder loading. What the classifier changes there is how often the reallocation happens and how early, and the sensitivity analysis of Section 7.6 is the closest available evidence on how much that matters, since moving the class cut-points moves lead time and severe-class recall while leaving the strategies untouched.

## 8. Discussion

### 8.1. Sensor Fusion and Feature Importance

That sEMG heads the SHAP ranking and carries the largest ablation decrement is expected; neuromuscular activation is the quantity closest to the physiology being estimated, and no other non-invasive modality gets nearer. Two results in Section 7.4 are less obvious, and both bear on how a system of this kind should be built.

Consider first the size of the contextual contribution. Removing three variables that a manufacturing execution system already logs costs 5.0 points of balanced accuracy, more than removing the entire six-unit inertial array, and elapsed time since the last break ranks fourth in the SHAP attribution. Fatigue is in large part accumulated exposure, and exposure is a quantity that a factory measures for free as a by-product of scheduling. A tiered deployment follows. A plant can begin with contextual features alone, which reach 63.7% balanced accuracy against a chance level of 33.3%, at essentially zero hardware cost. That figure is far too low to trigger an intervention, but it is high enough to show which stations and which work shift patterns justify instrumentation, and the sEMG channels can then be added where the coarse signal indicates that they are warranted. This inverts the usual order, in which the full sensor suite is installed first and its value argued afterwards.

The second concerns the extent of redundancy between modalities. The sub-additivity in Table 7 is pronounced: sEMG alone and everything-but-sEMG give almost the same result—79.8% against 80.1%—and the union of sEMG and IMU falls 4.5 points short of the full vector. Fatigue expresses itself simultaneously in the myoelectric spectrum, in movement smoothness, and in grip control, so each modality is partially a proxy for the others. For fusion research, this is a caution against reading multi-modal gains as evidence of complementary information; for a plant engineer, it is encouraging, since it means that performance degrades less when channels are removed than their number would suggest. The 31-dimensional configuration of Section 7.4 makes the point concretely: the device count falls from 18 to 4 for 3.1 points of balanced accuracy.

### 8.2. Architecture Selection and Real-Time Trade-Offs

Three of the eight architectures are statistically indistinguishable from the 1D-CNN–LSTM hybrid, and one of them—the CNN–BiLSTM–attention model—is nominally 0.3 points better. Accuracy is therefore not the criterion that separates them, and it should not be presented as though it were. The hybrid is preferable in terms of cost: it is the smallest neural model in the comparison, its arithmetic cost is the lowest of the five sequential architectures, and its 0.28 MB footprint quantized to 0.09 MB would fit on a microcontroller-class gateway that none of the alternatives would. Against the attention model specifically, the hybrid gives up 0.3 accuracy points, and in return it is 1.6 times smaller and 1.8 times faster.

The hybrid has a second, non-quantitative advantage: its convolutional front end learns filters that correspond to recognizable temporal events, sEMG bursts and movement onsets among them, and those filters can be plotted and shown to an ergonomist, which a recurrent state cannot. Where that kind of inspection is not required, the plain LSTM at 88.5% is simpler still, and nothing important is lost.

Two negative results are recorded here; passing over them would misrepresent what the comparison showed. The Transformer encoder gained nothing over the recurrent models despite four times the parameters of the hybrid, because a six-position sequence gives self-attention almost nothing to weight; its attention maps were near-uniform in every fold. Investigators tempted to apply a Transformer to this problem should either lengthen the sequence substantially or expect the same outcome. The TCN, by contrast, was the most efficient sequential model on this hardware, matching the LSTM to within 0.3 accuracy points at 16 ms, and it is the architecture to prefer in any deployment whose binding constraint is latency; when model size is the most important constraint, the hybrid remains preferable.

The architecture comparison is where the end-to-end latency figure is most likely to be misread. What the architecture controls is inference, at 8 to 39 ms across the eight models, and none of the 87–127 ms of Section 3.2 would move appreciably if a different one were selected.

XGBoost has a stronger case than a baseline model usually does. It reaches 86.7% at 15 ms, needs no sequence formatting, and trains and runs on a CPU. For a retrofit into an existing cell, where the constraint is what can be installed alongside equipment already in place, the 2.6-point deficit in accuracy is likely to be the right price for eliminating the GPU and the deep learning toolchain.

### 8.3. Personalization and Inter-Operator Variability

The 34% spread in endurance time constants translates into an accuracy range of 85.1–92.8%. The mechanism behind it is straightforward. An operator who transitions quickly spends less time in intermediate states and, therefore, contributes fewer windows near the moderate-to-severe boundary, and those are exactly the examples that the classifier needs. Fine-tuning on accumulating per-operator data would address this directly and remains untested here.

Per-operator calibration repays its 30 min of production time several times over. A generic population model, trained without individual $T_{i}$ identification and evaluated on the same leave-operators-out partition, reached 73% against 89.3% for the calibrated version. That is 16.3 percentage points for half an hour per operator, once. It also answers the deployment question raised in Section 6.1: matching an operator to the nearest pre-existing synthetic model on anthropometry alone recovers only part of that gap, reaching 81.4% in a leave-operators-out test in which the held-out operator was assigned the $T_{i}$ of its nearest anthropometric neighbor. Anthropometric matching is therefore a reasonable arrangement for the first day of use, but it is no substitute for per-operator calibration thereafter. The result sits comfortably within the precision-medicine argument now being made in occupational health more broadly [99].

### 8.4. In Silico Validation: Strengths and Limitations

Three things are gained by validating in silico: no human is deliberately driven into an injurious loading regime; the psychological, motivational, and contextual confounders that make occupational fatigue studies hard to interpret are absent, so a biomechanical relationship can be isolated; and the ground truth is exact by construction, rather than being inferred from a sensor proxy or taken from an operator’s self-report.

The corresponding limitations are equally definite, and they bound what the numbers in Section 7 can be taken to mean. A synthetic operator has no pain perception and no tolerance for it, no learned pacing strategy, no awareness of a supervisor or a production incentive, and no memory of yesterday’s work shift, so multi-day accumulation under insufficient recovery—central though it is to how chronic disorders actually develop—falls outside the model entirely [100]. The synthetic signals also inherit the noise model written into the generator: Gaussian additive noise and stochastic packet loss are a reasonable first approximation, but they do not reproduce electrode lift-off, perspiration-driven impedance drift over a work shift, or the non-stationary interference spectra of a real work floor.

Three consequences follow, and they are stated here in the form that the reviewer of a deployment proposal would expect:

First, the numerical results are properties of this simulation. The 89.3% accuracy, the 43% load reduction, and the 12 min lead time are not predictions about a factory, and they should not be cited as though they were. What the digital twin does establish is that the pipeline runs end-to-end inside its latency budget, that the eight architectures rank in a particular order, and that certain design trade-offs exist among accuracy, sensor count, and threshold placement. Clinical effectiveness lies outside the scope of a digital twin study.

Second, the ranking of the architectures is more transferable than their absolute accuracies, because it depends on the temporal structure of the fatigue trajectory, on which the fidelity of the signal generator has no bearing, and that structure is imposed by Equation (5), which was fitted to human endurance data. The ablation ordering is more transferable still, since it rests on which physical quantity carries the information, not on how well any of them is simulated. The absolute accuracies are the least transferable quantity in the paper.

Third, what a human study would have to measure is now specific. It would need concurrent sEMG, inertial, and force recording under a shift-length assembly protocol; a fatigue reference independent of the sensors, most plausibly periodic MVC testing supplemented by Borg CR-10 ratings [101]; and enough participants for a leave-operators-out design to be meaningful. The quantity to report is the correlation between the FDI trajectory computed by the biomechanical model and the measured decline in MVC, since that is the link that this study assumes and cannot test.

A limitation of a different kind attaches to the intervention trials. Their two arms differ by the whole policy, prediction and fixed thresholds together, so the 43% and the 6% are attributes of that policy and not of the classifier within it. Section 7.9 says how far the two can be told apart without the threshold-only arm that was never run.

One further caveat concerns the anthropometry. ANSUR-II describes a U.S. military population, and body proportion distributions elsewhere differ enough that the cohort should not be treated as globally representative. Beyond anthropometry, work–rest preferences and willingness to report discomfort vary culturally in ways that will shape adoption regardless of how the system performs technically.

### 8.5. Comparison to Prior Approaches and Integration Perspective

Placing 89.3% against published figures requires care, because the comparison is only meaningful where the evaluation protocol is the same, and the present figure was obtained on synthetic data whereas the others were not. Hwang et al. reported 87.94% with a CNN–LSTM–attention model on sEMG and IMU under leave-one-subject-out cross-validation [26], which is the closest match to the protocol used here; the present system adds force sensing and model-derived biomechanical features and obtains a marginally higher figure, and the difference is small enough that it should be attributed to the data, not to the method. Manni et al. reached 95.6% on a three-level task using transfer learning applied to continuous wavelet scalograms [102], but on a different population—elderly and non-elderly adults in an assisted-living context—with self-assessed labels and a different signal representation, so the higher figure does not indicate a better method for the present problem. Mohapatra et al. cannot be compared numerically at all: their target is a continuous perceived-exertion score and their metric is mean absolute error, roughly 2.3 on a 0–10 scale for non-overlapping users [31]. What their study does supply, and this one cannot, is data from real workers on real factory floors.

This study also stands in a particular structural relationship to its predecessor, and the relationship should be spelled out. A prior simulation study addressed the reallocation problem itself, showing that redistributing assembly subtasks between operator and cobot lowers cumulative musculoskeletal exposure without breaking production continuity [103]. That study assumed that the fatigue state was known; the present one supplies it. A reallocation algorithm without a state estimate has no input on which to act, and a state estimate without a reallocation algorithm has no output that changes anything. Joining the two, so that the prediction output feeds an allocator optimizing jointly over ergonomic exposure, throughput, and robot utilization, is the obvious next step and is not attempted here.

### 8.6. Economics of the Throughput Trade-Off

Whether a 6% throughput cost is acceptable is a question that this study cannot settle, since the answer depends on margin, staffing, and insurance arrangements that vary between facilities. What can be said is that fatigue-related productivity loss is itself a substantial cost at the sector level [31], so the comparison is not between a cost and no cost, but between two costs, only one of which currently appears on a production report. The 88% reduction in injury-risk events would have to be converted into an expected reduction in claims, lost days, and turnover before the two sides could be placed on the same scale, and that conversion requires local injury rates and local claim costs. A facility-specific analysis is therefore a prerequisite, not a refinement, and nothing in Table 10 should be read as a general recommendation to accept the trade-off.

### 8.7. Practical Deployment Considerations

The instrumentation described in Section 3 is not, as specified, something that a plant would accept, and treating that as a secondary limitation would misrepresent it. This is the primary obstacle between this study and a deployment, and the reduced configurations analyzed in Section 7.4 exist because of it. Five distinct problems have to be addressed:

*Donning Time and Wearer Compliance:* Fourteen body-worn sensors take an estimated 25 min to fit, every work shift, and the estimate assumes a trained assistant. No level of predictive accuracy matters if the operator has concluded that the setup is not worth the trouble. Three mitigations are available, and they are complementary rather than mutually exclusive: textile-integrated electrodes, which replace the placement of individual electrodes with the single act of putting on a garment [104,105]; quick-release magnetic mounts for the inertial units; and, most consequentially, the reduced 31-dimensional configuration of Table 7, which uses two electrodes and two inertial units on the dominant arm and retains 84.0% balanced accuracy. Fitting four sensors instead of fourteen brings donning inside the 5–8 min that a work shift changeover can absorb.

*Signal Drift over a Work Shift:* Perspiration lowers skin–electrode impedance initially and then destabilizes it as the gel layer is displaced, and the effect over an eight-hour work shift is not a fixed offset but a slow drift in both amplitude and noise floor [106]. Because the fatigue index of Equation (1) is normalized against a session baseline, a monotone drift is partially absorbed, but it is partially confounded with the very trend that the system is trying to detect, since both raise amplitude over time. Two countermeasures are indicated: One is periodic re-baselining during scheduled breaks, when activation is known to be low, which re-anchors ${\mathrm{MDF}}_{i}\left(t_{0}\right)$ without interrupting production. The other is continuous electrode–skin impedance monitoring, which most current wireless sEMG units already provide, used as a quality flag that suppresses the alert, never as an input to the classifier. Movement artifacts from cable-free sensors are a smaller problem than they once were, although impacts remain, and the saturation and dropout flags of Section 3.3 already remove the affected windows at a cost of 8.6% of the data.

*Maintenance and Consumables:* Gelled Ag/AgCl electrodes are single-use and, at eight sites per operator per work shift, a 50-operator plant consumes roughly 100,000 electrodes a year. Dry or textile electrodes remove that consumable at the cost of a higher and less stable contact impedance, which is the trade-off that the reduced configuration makes more tolerable, since two sites can be given better mechanical support than eight. Inertial units need periodic magnetometer recalibration in an environment containing three servo-driven manipulators, and the FSR elements—being bonded to tools instead of worn—follow the tool maintenance schedule and drift with mechanical wear of the handle.

*Equipment Cost:* At list prices, the full platform—eight research-grade sEMG sensors, six inertial units, four force elements, gateway, and edge compute—is a capital item of the order of tens of thousands of US dollars per instrumented station, and that is difficult to justify per operator. The reduced configuration cuts the sensor count by more than two thirds, and the tiered strategy of Section 8.1 cuts it further by instrumenting only the stations that contextual monitoring flags. Consumer-grade sEMG hardware is an order of magnitude cheaper and has already been shown to track subjective exertion in workplace trials [48], albeit at a signal quality that the spectral features used here have not been tested against.

*Electromagnetic Compatibility:* The digital twin is of little help here, since the interference model that it uses is a simplification, and real spectra near welding stations, high-power motors, and drive electronics are neither Gaussian nor stationary. A site survey before deployment is not optional, and its results should determine both the filtering strategy and where sensors can usefully be placed.

A sixth obstacle is not technical at all. Continuous physiological monitoring of employees generates data that can be used for purposes other than the one for which it was collected, and workers know this. Sustaining participation requires that data use be stated plainly, that aggregation genuinely prevent individual performance tracking, and that applicable data protection law be complied with rather than merely invoked. The human-centric framing of Industry 5.0 places this among the design requirements, not among the afterthoughts [107].

## 9. Conclusions

This paper sets out a wearable platform and a learning pipeline for continuous fatigue estimation in human–robot collaborative assembly, and it evaluates both in silico, in a digital twin spanning 1536 simulated operational hours. Eight sEMG channels, six IMUs, and four FSRs feed a 127-dimensional descriptor computed over 30 s windows at 15 s intervals.

Of the eight architectures compared under leave-operators-out cross-validation, the 1D-CNN–LSTM hybrid gave the best combination of accuracy and cost: 89.3% accuracy at 22 ms and 73.9 k parameters. Three sequential models, including a Transformer encoder and a CNN–BiLSTM–attention network, were statistically indistinguishable from it, and the nominally best of them paid 1.6 times the parameters and 1.8 times the latency for those 0.3 points. The durable finding therefore concerns temporal context: all five sequential models beat all three instantaneous ones, and every difference across that boundary was significant while none within the sequential group was.

Ablation attributed 7.0 points of balanced accuracy to the sEMG block and 5.0 points to three contextual variables that require no sensor, and it showed that a reduced configuration of two electrodes, two inertial units, and those contextual variables retains 84.0%. SHAP attribution ranked the sEMG fatigue index and cumulative shoulder loading highest, with elapsed time since the last break in fourth place. Sensitivity analysis over the fatigue class cut-points changed balanced accuracy by 2.5 points and left the architecture ranking intact, while severe-class recall and alert lead time moved in the opposite direction to accuracy; the operating point was therefore selected on lead time.

In the intervention trials, alert-triggered reallocation cut peak shoulder moment by 43% and injury-risk events by 88%, at a cost of 6% throughput, with alerts arriving about 12 min before the severe fatigue state.

Every one of these figures characterizes a simulation. They establish that the pipeline is internally coherent and fast enough, and they identify the design trade-offs among accuracy, sensor count, threshold placement, and computational cost. They do not establish clinical effectiveness. The synthetic operators lack pain perception, learned pacing, and multi-day recovery dynamics, all of which would bear on a real result, and the correlations reported between the simulated fatigue index and the sensor-derived indicators demonstrate internal consistency; they cannot establish external validity. The intervention figures carry one further qualification, of attribution rather than of realism: the 43% and the 6% belong to the classifier and the two fixed thresholds jointly, and a threshold-only arm would be needed to say how much of either the prediction earns. Prospective validation with human participants (approved by an institutional review board, n≥20, over 2–4 weeks, with periodic MVC testing as an independent fatigue reference) is the necessary next step. Multi-site testing should follow, and then the coupling of the prediction output to the task allocation layer discussed in Section 8, so that the loop from measurement to intervention closes without a human in the middle of it.

## Figures and Tables

**Table 1:** Wearable sensor platform specifications. Sampling rates differ by an order of magnitude across modalities, and this disparity is what the synchronization step of Section 3.2 must resolve.

Modality	Device	Units ^a^	Rate (Hz)	Channels ^b^	Quantity Measured
sEMG	Trigno Avanti (Delsys Inc., Natick, MA, USA)	8	1000	8	Muscle activation
IMU	MTw Awinda (Xsens Technologies B.V., Enschede, The Netherlands)	6	100	54	9-axis segment motion
FSR	FlexiForce A201 (Tekscan, Inc., Norwood, MA, USA)	4	500	4	Contact force
**Total**		**18**		**66**	

Bold marks the totals row, which sums the rows above it. ^a^ Physical devices worn or mounted. ^b^ Raw data channels each device produces: one per sEMG sensor, nine per IMU (three accelerometer, three gyroscope, and three magnetometer axes), and one per FSR element. Abbreviations: sEMG, surface electromyography; IMU, inertial measurement unit; FSR, force-sensitive resistor.

**Table 2:** Composition of the 127-dimensional feature vector extracted from each 30 s window. The third column gives the number of scalar features that each category contributes to a single window.

Category	Descriptors	Features per Window	Origin
sEMG, time domain	RMS, MAV, WL, ZC, SSC	40	8 sEMG channels
sEMG, frequency domain	MDF, MNF, fatigue index	24	8 sEMG channels
IMU kinematics	Angles, velocities, jerk, ROM	36	6 IMU units
Biomechanical	Torques, loads, mechanical work	12	Inverse dynamics
FSR force dynamics	Peak, mean, variability, rate	12	4 FSR elements
Contextual	Elapsed times, cycle count	3	MES
**Total **		**127**	

Bold marks the totals row, which sums the rows above it. Abbreviations: RMS, root mean square; MAV, mean absolute value; WL, waveform length; ZC, zero crossings; SSC, slope sign changes; MDF, median frequency; MNF, mean power frequency; ROM, range of motion; MES, manufacturing execution system.

**Table 3:** Synthetic operator model specifications (n=24). Parameters derived from the ANSUR-II anthropometric database and U.S. manufacturing workforce demographics.

Characteristic	Mean ± SD	Range	KS *D* ^a^	*p*
Age (years)	38.4±9.2	24–56	—	—
Stature (cm)	167.3±8.4	152–184	0.08	0.62
Body mass (kg)	71.2±11.8	54–96	0.09	0.54
BMI (kg m^−2^)	25.4±3.1	20.3–31.2	0.07	0.71
Endurance $T_{i}$ (s) ^b^	142±48	85–218	—	—

The cohort is sex-balanced: 12 male and 12 female models. ^a^ Two-sample Kolmogorov–Smirnov statistic against the ANSUR-II reference distribution; a non-significant *p* indicates that the sampled cohort is not distinguishable from the target population on that variable. ^b^ Endurance time constant of Equation (5); the symbol $T_{i}$ is used throughout in place of the conventional τ, which denotes the joint torque vector in Equation (3). Abbreviations: SD, standard deviation; BMI, body mass index; KS, Kolmogorov–Smirnov.

**Table 4:** Fatigue classification performance of eight model architectures, pooled over the six out-of-fold test partitions (24 operators, 294,912 windows). The models are grouped by whether a decision uses one feature vector or a sequence of six.

Model	Accuracy	95% CI	Balanced	Macro F1	AUC	Latency	*p* vs.
	(%)	(%)	Accuracy (%)	(%)		(ms)	Hybrid ^a^
*Instantaneous models: one feature vector per decision*							
SVM (RBF)	82.1	80.3–83.9	79.4	80.0	0.89	**8**	<0.001
Random Forest	84.3	82.6–86.0	81.7	82.3	0.91	12	<0.001
XGBoost	86.7	85.1–88.3	84.2	85.0	0.93	15	0.002
*Sequential models: six consecutive feature vectors per decision*							
TCN	88.2	86.6–89.8	85.9	86.6	0.94	16	0.088
LSTM	88.5	86.9–90.1	86.3	87.0	0.94	18	0.194
Transformer	88.9	87.2–90.6	86.6	87.3	0.94	27	0.471
1D-CNN–LSTM	89.3	87.8–90.8	87.1	87.7	0.95	22	—
CNN–BiLSTM–Attn.	**89.6**	88.0–91.2	**87.4**	**88.0**	**0.95**	39	0.612

Bold indicates the best value in a column. Confidence intervals are from an operator-level cluster bootstrap (B=1000 resamples over the 24 operators). ^a^ Two-sided Wilcoxon signed-rank test on the 24 per-operator balanced accuracies, taking the 1D-CNN–LSTM hybrid as the reference model. Every difference across the boundary between the two families is significant at p<0.01; no difference within the sequential family is significant. Abbreviations: SVM, support vector machine; RBF, radial basis function; TCN, temporal convolutional network; LSTM, Long Short-Term Memory; CNN, convolutional neural network; BiLSTM, bidirectional LSTM; Attn., attention; CI, confidence interval; AUC, area under the receiver operating characteristic curve.

**Table 5:** Computational cost of the eight architectures on the edge target of Section 4. Latency alone understates deployment cost, so parameter count, arithmetic cost, serialized size, peak memory, and energy are reported alongside it.

Model	Parameters ^a^	FLOPs	Size	Peak RAM	Latency	Energy
		(M) ^b^	(MB) ^c^	(MB)	(ms)	(mJ)
*Instantaneous models*						
SVM (RBF)	21,483 SVs	5.46	10.90	96	**8**	**28**
Random Forest	1.28 M nodes	0.06	24.60	118	12	41
XGBoost	0.21 M nodes	0.02	4.10	74	15	47
*Sequential models*						
TCN	118.9 k	1.42	0.45	62	16	58
LSTM	180.7 k	2.17	0.69	68	18	66
Transformer	281.1 k	3.38	1.07	81	27	103
1D-CNN–LSTM	**73.9 k**	0.69	**0.28**	**57**	22	79
CNN–BiLSTM–Attn.	117.2 k	1.16	0.45	66	39	148

Bold indicates the lowest cost in a column. Latency, peak memory, and energy are medians of 1000 single-sample inferences on an NVIDIA Jetson Nano in 10W mode, after 100 warm-up runs and with the clocks pinned. ^a^ Trainable parameters for the five neural models; for the tree ensembles and the kernel machine, the corresponding structural quantity is given instead, since neither has trainable parameters in the same sense. ^b^ Multiply–accumulate operations per single-sample inference, counted analytically. ^c^ Serialized 32-bit floating-point model; post-training INT8 quantization reduces the hybrid to 0.09 MB and 14 ms. Abbreviations: SVs, support vectors; FLOPs, floating-point operations; RAM, random-access memory.

**Table 6:** Per-class performance of the 1D-CNN–LSTM hybrid, computed from the pooled out-of-fold confusion matrix of Figure 5a. Support is the number of windows belonging to each true class.

Fatigue Class	Precision (%)	Recall (%)	F1 (%)	Support (Windows)
Fresh (FDI ≤40)	92.8	93.5	93.1	132,708
Moderate (40< FDI ≤70)	86.7	89.1	87.9	112,068
Severe (FDI >70)	85.8	78.6	82.0	50,136
**Macro average **	**88.4**	**87.1**	**87.7**	**294,912**

The macro-averaged recall is the balanced accuracy reported in Table 4; the macro-averaged F1 is the value reported there as Macro F1. Overall accuracy over the same predictions is 89.3%. Abbreviation: FDI, Fatigue Demand Index.

**Table 7:** Modality ablation for the 1D-CNN–LSTM hybrid over fifteen feature configurations. The last column gives the wearable burden that each configuration imposes, which is the quantity by which a deployment is constrained.

Feature Configuration	Dimensions	Balanced	Δ (pp) ^a^	Body-Worn
		Accuracy (%)		Sensors ^b^
Full vector	127	87.1	—	14
*Leave-one-block-out*				
Without sEMG	63	80.1	−7.0	6
Without contextual	124	82.1	−5.0	14
Without IMU ^c^	91	83.4	−3.7	8
Without biomechanical	115	83.6	−3.5	14
Without FSR	115	83.9	−3.2	14
Without IMU and biomechanical	79	81.2	−5.9	8
*Single block*				
sEMG only	64	79.8	−7.3	8
IMU only	36	71.3	−15.8	6
Contextual only	3	63.7	−23.4	0
FSR only	12	58.4	−28.7	0
*Combinations*				
sEMG + IMU	100	82.6	−4.5	14
sEMG + contextual	67	83.2	−3.9	8
sEMG + IMU + contextual	103	85.4	−1.7	14
*Reduced deployment set*				
2 sEMG + 2 IMU + contextual	31	84.0	−3.1	4

Every configuration was retrained from scratch with re-tuned hyperparameters under the same six-fold leave-operators-out rotation. Chance level for three classes is 33.3%. ^a^ Change in balanced accuracy relative to the full 127-dimensional vector, in percentage points. ^b^ Number of sensors that the operator has to wear; the four FSR elements are mounted on tools and on the workstation and are therefore not counted. ^c^ The biomechanical block is derived from the IMU and FSR signals, so this row is not a configuration that a physical deployment could realize; the row that removes both blocks is reported alongside it for that reason.

**Table 8:** Sensitivity of the results to the FDI cut-points that define the three fatigue classes. Balanced accuracy, severe-class recall, and alert lead time move in opposite directions across the range, so the three do not share an optimum.

FDI Cut-Points	Class Balance	Balanced	Severe	Severe	Lead Time
(Lower, Upper)	(%) ^a^	Accuracy (%)	Recall (%)	F1 (%)	(min) ^b^
(30, 60)	31/41/28	85.9	84.1	85.0	18.4
(35, 65)	38/40/22	86.5	81.7	83.6	15.1
**(40, 70)** ^c^	45/38/17	**87.1**	**78.6**	**82.0**	**12.0**
(45, 75)	52/35/13	87.6	76.2	79.9	9.4
(50, 80)	59/32/9	88.4	71.8	76.3	7.6

Each row is a complete relabeling of all 294,912 windows, followed by retraining and re-evaluation of the 1D-CNN–LSTM hybrid under the same six-fold rotation, with class weights recomputed for the new balance. ^a^ Percentage of windows in the fresh, moderate, and severe classes, respectively. ^b^ Mean interval between the first alert and the crossing into the severe fatigue class. ^c^ Setting adopted in the rest of the paper; bold marks that row, not the best value in each column. Balanced accuracy is highest at (50, 80), where severe-class recall and lead time are worst, which is why the operating point was not selected on accuracy.

**Table 9:** Association between the simulated Fatigue Demand Index and seven sensor-derived fatigue indicators, ordered by the magnitude of the association.

Indicator	Modality	Spearman ρ	95% CI	Expected Sign ^a^
Biceps brachii fatigue index ^b^	sEMG	+0.86	[+0.82, +0.89]	positive
Cumulative shoulder load	Biomechanical	+0.83	[+0.79, +0.87]	positive
Biceps brachii median frequency	sEMG	−0.81	[−0.85, −0.76]	negative
Anterior deltoid median frequency	sEMG	−0.78	[−0.83, −0.72]	negative
Biceps brachii RMS amplitude	sEMG	+0.74	[+0.68, +0.79]	positive
Normalized jerk, dominant upper arm	IMU	+0.61	[+0.53, +0.68]	positive
Grip force coefficient of variation	FSR	+0.57	[+0.47, +0.65]	positive

Spearman ρ computed within operator over all windows of that operator, and then pooled across the n=24 operators by Fisher *z* transformation; intervals are 95% confidence intervals on the pooled estimate. sEMG quantities are taken from the dominant side. Every observed sign agrees with the electrophysiological expectation. ^a^ Direction predicted by the fatigue literature for a rising fatigue state. ^b^ As defined in Equation (1). These coefficients establish internal consistency only: the Fatigue Demand Index and the sEMG descriptors are both outputs of the same generative model, so no claim about human physiology follows from them (Section 8.4).

**Table 10:** Effect of alert-triggered task reallocation on musculoskeletal loading and on throughput; baseline versus prediction-triggered condition.

Outcome	Baseline	Prediction-	Change	Cohen’s	*p* ^a^
(Unit)	(Mean ± SD)	Triggered	(%)	*d*	
Peak shoulder moment (N m)	18.3±4.2	10.4±2.8	−43	2.21	<0.001
Cumulative load (N m·h)	12.7±3.1	8.8±2.4	−31	1.41	<0.001
Injury-risk events per work shift	3.2±1.4	0.4±0.6	−88	2.58	<0.001
Fatigue Demand Index (0–100)	61.2±12.3	40.7±9.8	−34	1.84	<0.001
Throughput (assemblies per hour)	7.8±0.9	7.3±0.8	−6	0.59	0.012

Estimates from mixed-effects models with operator as a random intercept, over 192 sessions (96 baseline, 96 prediction-triggered) in a within-operator crossover design. ^a^ Bonferroni-corrected for the five comparisons. The throughput reduction is small but statistically detectable and is reported as such; whether it is acceptable is a question of plant economics and is taken up in Section 8.6. Abbreviation: SD, standard deviation.

## Data Availability

All code and simulation resources supporting this study are publicly available, in both cases under CC-BY-4.0, to permit independent replication and to comply with the FAIR (Findable, Accessible, Interoperable, Reusable) principles. Source code is hosted on GitHub at https://github.com/ClaudioUrrea/CNNLSTM-Wearable-FatiguePrediction (accessed on 30 August 2026), and the archived release, cohort specification, and trained models are deposited on Figshare at https://doi.org/10.6084/m9.figshare.33095672. The deposit comprises the CoppeliaSim scene and Lua control scripts implementing the 23-DOF digital human, the synthetic sensor generation code for all three modalities, the specification of the 24 synthetic operators in CSV format, the random seeds governing every stochastic component, the trained weights of the eight architectures for each of the six folds, the ablation and threshold-sensitivity configurations, per-operator out-of-fold accuracies, the figure generation script (Python 3 with NumPy 1.24 or later and Matplotlib 3.7 or later, as declared in the deposited environment file), and an automated audit that recomputes every quantity reported in this article and fails on any disagreement. The 294,912 feature windows analyzed in Section 7 are not archived as an array: they are a deterministic function of the deposited configuration and seed set, so regeneration is exact and also allows a reader to perturb the inputs and observe the effect on the conclusions. The figure generation script reads every plotted quantity from one table of results and recomputes the per-class precision, recall, and F1 values, the balanced accuracy, the macro F1, and the severe-class operating point directly from the confusion matrix, halting if any of them disagrees with the corresponding table in this article. No figure can therefore fall out of agreement with a table without the script failing. Installation instructions, the experimental protocol, and a data dictionary accompany the repository. No data of human origin were collected at any stage, so nothing in the archive is restricted.

## Abbreviations

The following abbreviations are used in this manuscript:

ANSUR	Anthropometric Survey of U.S. Army Personnel
AUC	Area Under the Curve
BiLSTM	Bidirectional Long Short-Term Memory
BLE	Bluetooth Low Energy
BMI	Body Mass Index
CNN	Convolutional Neural Network
CI	Confidence Interval
DOF	Degrees of Freedom
FDI	Fatigue Demand Index
FI	Fatigue Index
FLOPs	Floating-Point Operations
FSR	Force-Sensitive Resistor
GPU	Graphics Processing Unit
HRC	Human–Robot Collaboration
HRV	Heart Rate Variability
IIR	Infinite Impulse Response
IMU	Inertial Measurement Unit
KS	Kolmogorov–Smirnov
LSTM	Long Short-Term Memory
MAV	Mean Absolute Value
MDF	Median Frequency
MES	Manufacturing Execution System
MFLOPs	Million Floating-Point Operations
ML	Machine Learning
MNF	Mean Power Frequency
MVC	Maximum Voluntary Contraction
NASA-TLX	NASA Task Load Index
RBF	Radial Basis Function
ROC	Receiver Operating Characteristic
REBA	Rapid Entire Body Assessment
RF	Random Forest
RMS	Root Mean Square
ROM	Range of Motion
RPE	Rating of Perceived Exertion
RULA	Rapid Upper Limb Assessment
sEMG	Surface Electromyography
SENIAM	Surface EMG for Non-Invasive Assessment of Muscles
SD	Standard Deviation
SHAP	SHapley Additive exPlanations
SNR	Signal-to-Noise Ratio
SSC	Slope Sign Changes
SVM	Support Vector Machine
TCN	Temporal Convolutional Network
WL	Waveform Length
WMSDs	Work-Related Musculoskeletal Disorders
XGBoost	eXtreme Gradient Boosting
ZC	Zero Crossings
